# A comprehensive systematic review of the development process of 104 patient-reported outcomes (PROs) for physical activity in chronically ill and elderly people

**DOI:** 10.1186/1477-7525-9-116

**Published:** 2011-12-20

**Authors:** Anja Frei, Kate Williams, Anders Vetsch, Fabienne Dobbels, Laura Jacobs, Katja Rüdell, Milo A Puhan

**Affiliations:** 1 Horten Centre for Patient-oriented Research, University Hospital of Zurich, Switzerland; 2 Institute of General Practice and Health Services Research, University Hospital of Zurich, Switzerland; 3 Patient Reported Outcomes Centre of Excellence, Global Market Access, Primary Care Business Unit, Pfizer Ltd, Walton Oaks, Surrey, United Kingdom; 4 Centre for Health Services and Nursing Research, post-doctoral researcher FWO Vlaanderen, Katholieke Universiteit Leuven, Leuven, Belgium; 5 Respiratory Sciences, Katholieke Universiteit Leuven, Leuven, Belgium; 6 Department of Epidemiology, Johns Hopkins Bloomberg School of Public Health, Johns Hopkins University, Baltimore (MD), USA

## Abstract

**Background:**

Capturing dimensions of physical activity relevant to patients may provide a unique perspective for clinical studies of chronically ill patients. However, the quality of the development of existing instruments is uncertain. The aim of this systematic review was to assess the development process of patient-reported outcome (PRO) instruments including their initial validation to measure physical activity in chronically ill or elderly patient populations.

**Methods:**

We conducted a systematic literature search of electronic databases (Medline, Embase, Psychinfo, Cinahl) and hand searches. We included studies describing the original development of fully structured instruments measuring dimensions of physical activity or related constructs in chronically ills or elderly. We broadened the population to elderly because they are likely to share physical activity limitations. At least two reviewers independently conducted title and abstract screening and full text assessment. We evaluated instruments in terms of their aim, items identification and selection, domain development, test-retest reliability, internal consistency, validity and responsiveness.

**Results:**

Of the 2542 references from the database search and 89 from the hand search, 103 full texts which covered 104 instruments met our inclusion criteria. For almost half of the instruments the authors clearly described the aim of the instruments before the scales were developed. For item identification, patient input was used in 38% of the instruments and in 32% adaptation of existing scales and/or unsystematic literature searches were the only sources for the generation of items. For item reduction, in 56% of the instruments patient input was used and in 33% the item reduction process was not clearly described. Test-retest reliability was assessed for 61%, validity for 85% and responsiveness to change for 19% of the instruments.

**Conclusions:**

Many PRO instruments exist to measure dimensions of physical activity in chronically ill and elderly patient populations, which reflects the relevance of this outcome. However, the development processes often lacked definitions of the instruments' aims and patient input. If PROs for physical activity were to be used in clinical trials more attention needs to be paid to the establishment of content validity through patient input and to the assessment of their evaluative measurement properties.

## Background

Physical activity is crucial to chronically ill patients' functioning in daily life. The evidence of the protective role of physical activity for the prevention and management of chronic diseases has been well established over recent decades [1,2]. Physical activity is a multidimensional construct and defined as "any bodily movement produced by the contraction of skeletal muscle that increases energy expenditure above a basal level" [3].

The assessment of physical activity as an outcome measure provides a unique perspective in chronic disease research not only for observational studies, but also for drug and nondrug clinical trials. Furthermore, evidence from trials regarding physical activity as a patient-reported outcome (PRO) could inform patients about treatment options that address relevant aspects of their daily life. Investigators who are interested in measuring physical activity face the challenge of not only choosing an instrument that serves their study aim, but that has also been carefully developed and validated. These instruments should have strong psychometric properties such as stability over time (test-retest reliability) and the capacity to detect even small effects (responsiveness to change). In addition, investigators need to be certain that the instruments reflect the dimensions of physical activity that are relevant to patients.

It is currently unclear whether available instruments to measure physical activity fulfil these requirements. Therefore, the aim of this systematic review, which is part of the Innovative Medicines Initiative PROactive project (http://www.proactivecopd.com a project jointly funded by the European Commission and the European Federation of Pharmaceutical Industries and Associations 'EFPIA), was to identify existing fully structured PROs (questionnaires, scales) measuring physical activity (frequency, intensity and total amount), and/or symptoms (physical and mental) or complaints/concerns associated with physical activity in chronically ill or elderly patient populations. We broadened the population to elderly because they are likely to share some characteristics regarding physical activity with chronically ill patients. Furthermore, the systematic review aimed to evaluate the methodological rigour with which the retrieved instruments were developed and initially validated as a part of the development process. Therefore, we restricted our review to the first validations of the instruments as part of the development process. In this paper we focused on the methods used in the development of the physical activity instruments. The content and the format of the included instruments are reviewed in another paper.

## Methods

A study protocol (unregistered) guided the entire review process. We followed standard systematic review methodology as outlined in the handbooks of the Centre for Reviews and Dissemination [4] and the Cochrane Collaboration. The reporting follows the PRISMA statement that recently replaced the former guidelines of reporting of systematic reviews and meta-analyses (QUOROM statement) [5].

### Eligibility criteria

We considered the following criteria for inclusion and exclusion:

#### Population

We included PRO instruments developed for patients with chronic disease or elderly people. Elderly people were included because chronic illnesses usually affect people in later stages of life. In addition, we supplemented the electronic database search with explicit search terms for COPD patients. This is because this systematic review is part of the PROactive project, which aims to develop and validate two PRO instruments for COPD patients [6].

#### Types of instrument

We included fully structured instruments (scales or questionnaires) with standardised questions and answer options which were reported by the patient (self-reported). We only included interviewer administered instruments if the information was self-reported by the patient and we excluded instruments that required a rating by an interviewer.

#### Content of instrument/assessment of physical activity

We included instruments measuring dimensions of physical activity or related constructs. We considered the following definition for physical activity according to the U.S. Department of Health and Human Services [3]: "Physical activity is defined as any bodily movement produced by the contraction of skeletal muscle that increases energy expenditure above a basal level". This definition of physical activity is broad and encompasses activities of daily living, sports and activities for personal fulfilment. We did not restrict the search to instruments measuring the frequency, intensity and total amount of physical activity, but also considered instruments assessing "related constructs" and/or subscales that focused on symptoms (physical and mental) or complaints/concerns associated with physical activity. All of the instruments we included contained at least one physical activity subscale. We only included instruments whose items we could access from the publication or from the developers. We did not have any language or publication date restrictions.

#### Study design

We included both cross-sectional and longitudinal studies which described the development (including item generation, piloting etc) or modifications of the original instrument and the initial validation (psychometric properties, cross-sectional or longitudinal) of the original instrument. Since we focused on the methods used for the development process of the instruments, the article had to describe a minimum of the development or first validation process, for example, a description about item identification or selection and/or at least one assessment of test-retest, responsiveness or validity in a publication that was clearly the original. We excluded studies that used an eligible instrument as an outcome measure and were not designed to initially validate this instrument. We also excluded studies that reported the validation of instruments in additional languages and/or populations.

### Information sources

#### Electronic database searches

We searched the electronic databases Medline, Embase, PsycINFO and CINAHL on September 18th 2009.

#### Hand searches

We conducted the following hand searches to complement the electronic database search results: We searched for original development studies of instruments from articles which were excluded for the reason "validation only" or "used as outcome measures"; we scanned the reference lists of the full texts; we searched the Patient-Reported Outcome and Quality of Life Instruments Database (PROQolid) on March 10 2010, search term: "physical functioning" questionnaires; and we contacted experts in the field and asked them to check if our list of included instruments was complete or if we missed any instruments.

### Search

For the electronic database search, we used the following search terms: (physical activity OR functioning OR function OR motor activity OR activities of daily living OR walking OR activity OR exercise) AND (questionnaire* OR scale OR instrument OR tool OR diary OR assessment OR self-report OR measure*) AND (valid*) AND (chronic disease OR elderly OR COPD OR chronic lung disease OR chronic obstructive lung disease) NOT (athletic performance OR sports OR children OR adolescent).

### Study selection

#### Title and abstract screening

Two pairs of two reviewers each used a title and abstract screening document to independently review the title and abstract of every article retrieved by the database search. Decisions to include or exclude were recorded in the RefWorks-COS file (0 = exclude, 1 = order for full text assessment, 2 = only validation study of existing instrument, 3 = related study (e.g. reviews), do not order but may be useful reference). We ordered all articles that were deemed potentially eligible by at least one reviewer.

#### Full text screening

Two pairs of two reviewers each independently evaluated the full texts and made a decision on inclusion or exclusion according to the predefined selection criteria. They recorded their decision on a paper form together with the reason for exclusion (not relevant patient group; instrument does not measure dimensions of physical activity or related constructs; instrument is not self-reported (e.g. functional or exercise test like time to stand up from a chair or 6 minutes walking test); instrument with all its items is not available from the publication or from the developers; instrument is used as an outcome measure/study is not designed to validate this instrument, respectively; validation study only (e.g. additional languages, populations etc.); other). If the two reviewers could not agree, a third reviewer decided whether to include or exclude. Studies that did not fulfil all of the predefined criteria were excluded and their bibliographic details were listed with the specific reason for exclusion.

#### Piloting the study selection process

Initially, all reviewers piloted the selection process by applying the inclusion and exclusion criteria to the 50 first references for titles and abstracts screening and the first 30 papers for full text assessment. Inclusion and exclusion criteria were refined and clarified based on this piloting process.

#### Dealing with lack of information

We made three attempts to contact authors by e-mail in the following conditions: 1) If it was unclear from the full text article whether the study fulfilled the inclusion and exclusion criteria; 2) If the included development study lacked information on how the instrument was developed in order to complete data extraction; 3) If the included development study lacked information on the instrument's content (items, introduction question, recall period etc.). If we failed to retrieve the relevant information from the author, this was reported on the data extraction form.

#### Dealing with duplicate publications

In cases where multiple papers were published (e.g. translations, reporting on different outcomes etc.), we treated the study with multiple reports as a single study but made reference to all publications.

### Data extraction process

We created standardised data extraction forms based on a form used in a previous review [7] to record the relevant information from the articles. The data extraction forms were piloted twice by four reviewers including 8 instruments for the first and 6 instruments for the second pilot. The forms and categories were then adapted and refined where necessary. The first reviewers extracted the data and stored it in a MS Word file. The second reviewers then independently extracted the data and compared their results with that of the first reviewers. These changes were made using the 'track changes' mode. The file was sent back to the first reviewer in order to come to an agreement. When an agreement could not be reached a third reviewer was consulted.

### Data extraction

We extracted data from the development studies regarding the instruments' development and initial validation process. We used pre-defined categories and answer options including numerical indications, fixed texts such as "yes/no", multiple choice and free text. We extracted data for the following categories:

#### Development of instruments

##### Aim of instrument

We distinguished between 3 categories: First, if the aim of the instrument was clearly described by the authors before the instrument was developed, the classification was "described". We differentiated between the four aims "evaluative" (detection of changes over time, typically for evaluation of treatments), "discriminative" (detection of differences between patients, e.g. for phenotyping), "predictive" (prediction of future health outcomes, e.g. hospital admissions or death) and "planning" (planning of treatment, e.g. detection of areas with low scorings to target patient education accordingly). Second, if the aim was not explicitly described by the authors before development but could be identified from the context, the classification was "not clearly described, but presumably (e.g. evaluative)". Third, if the purpose of the instrument was not reported and could not be identified we used the classification "not described".

##### Identification of items

To describe the identification of the items, we differentiated between five categories of sources of item generation (several answer options possible): patients and elderly (target population); experts (e.g. clinical experts, health professionals, care givers etc., also includes supplementation or modification of existing items through experts); significant others (e.g. family members, care givers); literature; and adaptation of existing instruments. We also described the method of item identification in brackets, for example, interviews or focus groups, systematic or unsystematic searches.

##### Selection of items

We reported the approach used by the authors to select items for the final instrument by differentiating between the following four sources: patients quantitative; patients qualitative; experts quantitative; experts qualitative. We provided specific details in brackets, for example, "Patients: quantitative (e.g. factor analysis)", "Patients: qualitative (e.g. focus group)", "Experts: quantitative (e.g. relevance)" or "Experts: qualitative (e.g. interviews)".

##### Development of domains

We recorded the method of how the domains were defined, i.e. if they were defined a priori (the authors predefined domains and items which belong to these domains without statistical analyses but based on their clinical/research experience or opinion) or if domains were statistically defined by factor analysis.

#### Initial validation of instruments

##### Test-retest

We recorded if test-retest reliability (reproducibility) was examined and described the statistical method used, for example, intra-class correlation coefficients, coefficient of variation, Pearson or Spearman correlation coefficients or t-tests.

##### Internal consistency

We recorded if internal consistency reliability was assessed and described the statistical method used, for example, Cronbach's alpha, corrected item total correlation or Cronbach's alpha excluding item analysis.

##### Validity

We recorded if validity was assessed and if so, the type of validity that the authors described to assess (in quotation marks) and the statistical methods used (in brackets).

##### Responsiveness

We recorded any approaches to assess responsiveness (i.e. the ability of an instrument to detect changes over time) and we reported the statistical methods used.

##### Minimal important difference (MID)

We reported if the MID was examined and the statistical methods (e.g. anchor- or distribution-based approaches) used.

##### Summary of conducted initial validation assessments according to aim of instrument

The aim of the instrument determines the measurement properties, which should be assessed in the validation process. The assessment of test-retest reliability and internal consistency is important for each instrument development, regardless of whether the instrument's aim is evaluative, discriminative, predictive or planning. For instruments with an evaluative aim, the longitudinal testing of the validity is of special interest whereas for instruments with discriminative or planning aims, cross-sectional testing of the validity is sufficient. For instruments with evaluative aims, the assessment of responsiveness and the MID is crucial because they aim to detect changes over time.

We summarised the assessed psychometric properties of the instruments for which the authors clearly described an aim before the instruments was developed.

### Synthesis of results

We described the results of the data extraction in structured tables according to the categories described above (see Additional file 1). We synthesised the data on the instruments' development and initial validation in a narrative way and in integrated tables. We used numbers and proportions to describe the results quantitatively. These frequencies were calculated using SPSS (Version 18.0).

## Results

### Study selection

**Figure 1:**
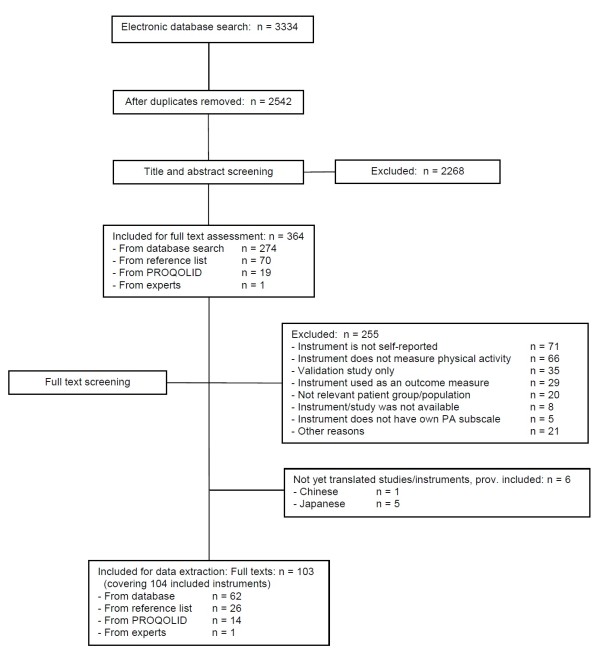
**Flow diagram of identification of studies**.

Figure 1 shows the flow diagram of the identification of the studies. The electronic database search produced 2542 references. After title and abstract screening, 2268 of these were excluded resulting in 274 articles for full text assessment. This included 5 Japanese and one Chinese language article which were provisionally included due to their English abstract but were not included in the current analysis as we were unable to translate them [8-13]. Hand searches of reference sections and of excluded articles revealed an additional 70 instruments/development studies for full text assessment. The search of the PROQolid database produced a further 58 instruments, 19 of which were included for full text assessment after title and abstract screening. One additional instrument was retrieved from the consultation with experts. Therefore, a total of 364 papers were included for full text assessment.

Following full text assessment, a further 255 were excluded resulting in 104 instruments from 103 full texts (the article of Mannerkorpi & Hernelid (2005) [14] provided information for the development process of two instruments) included in the review [14-117]. The most frequent reasons for exclusion were instrument is not self-reported (n = 71), followed by instrument does not measure physical activity (n = 66), validation study only (n = 35) and instrument used as an outcome measure (n = 29). The references of all excluded articles after full text assessment are summarised in Additional file 2.

### Study characteristics

Additional file 1 summarises the extracted data for the development and initial validation process of the reviewed instruments.

#### Aim of instrument

For almost half of the instruments (n = 49, 47.1%), the authors clearly described the aim of the instruments before the scales were developed. One aim was described for 26 instruments (53.1%) and more than one for 23 instruments (46.9%). The most frequently described aim was evaluative (n = 33), followed by discriminative (n = 26), planning (n = 13) and predictive (n = 5). For 43.3% of the instruments (n = 45), the authors did not clearly describe one or several aims but they could be presumed from the context (presumably discriminative: n = 32, presumably evaluative: n = 24, presumably planning: n = 9, presumably predictive: n = 9). For 10 instruments (9.6%), the authors did not describe an aim.

#### Identification of items

For 39 instruments (37.5%) items were identified with patient input, either with patient input only or with patient input together with other sources (adaptation of existing instruments, experts and/or literature). Adaptation of existing instruments and/or unsystematic literature searches only were the source for item identification of 33 instruments (31.7%), and expert input only or expert input additionally to literature and adaptation was the source for item identification of 14 instruments (13.5%). For the development of 18 instruments (17.3%), item identification was not reported or not clearly described. Table 1 describes the sources which were used to identify the items of the included instruments, ordered by frequency.

**Table 1:** Sources of item identification of the included instruments (n = 104)^1)^

Sources of item identification	n	%
Adaptation of existing scales only	18	17.3%
Patients & experts & literature (unsystematic search)^1)^	13	12.5%
Patients only	12	11.5%
Literature only (unsystematic search)	10	9.6%
Experts and literature (unsystematic search)	7	6.7%
Patients and literature (unsystematic search)	6	5.8%
Adaptation and literature (unsystematic search)	4	3.8%
Patients and experts	4	3.8%
Experts only	3	2.9%
Experts and adaptation and literature (unsystematic search)	2	1.9%
Patients and adaptation	2	1.9%
Adaptation and literature (systematic search)	1	1%
Adaptation and experts	1	1%
Patients and adaptation and literature (unsystematic search)	1	1%
Patients and experts and adaptation	1	1%
Significant others and literature (unsystematic search) and adaptation	1	1%
Not reported/not clearly described	18	17.3%

^1) ^For data extraction details, please see Additional file 1

The most frequently used method to generate patient input was "interviews with patients" only (for 24 of 39 instruments). Focus groups were less frequently conducted (for 5 of 39 instruments) and for only 1 instrument both interviews and focus groups were conducted. For 7 instruments, the method of generating patient input was not reported and for 2 instruments, patient input was described as "clinical interactions" or "open ended survey". The methods used to obtain expert input were more diverse and varied from interviews with experts to workshops, ratings of relevance, unspecified discussions and undefined consideration of clinical opinion. Literature searches were always conducted unsystematically.

#### Selection of items

For 58 instruments (55.8%), patient input was used for item reduction, and for 12 instruments (11.5%) the items were selected by expert input only. For 34 instruments (32.7%), item reduction was not clearly described (see Table 2). Where patient input was used for item selection (n = 58), the methods were predominantly quantitative (n = 31, 53.4%) and conducted by factor analysis (17 of 31 instruments). Less frequently used methods included item-total correlations, Rasch analyses and consideration of response rates and floor/ceiling effects. Qualitative methods, either alone or in addition to quantitative methods, were used in the selection of items for 46.6% (n = 27) of the instruments. Most frequently, qualitative patient input for item selection was generated by patient interviews (10 of 27 instruments). Less frequently focus groups and cognitive interviews/debriefings were used.

**Table 2:** Source and method for item selection of the included instruments (n = 104)^1)^

Source and method for item selection	n	%	n	%
**Selection with patient input**			**58**	**55.8%**
Patients quantitative	21	20.2%		
Patients qualitative	13	12.5%		
Patients qualitative and quantitative	6	5.8%		
Patients quantitative and experts qualitative	6	5.8%		
Patients and experts qualitative	5	4.8%		
Patients and experts quantitative	4	3.8%		
Patients and experts qualitative, patients quantitative	3	2.9%		
**Selection with expert input only**			**12**	**11.5%**
Qualitative	5	4.8%		
Quantitative	5	4.8%		
Quantitative and qualitative	2	1.9%		
**Not reported (n = 33) and N/A (n = 1)**			**34**	**32.7%**

^1) ^For data extraction details, please see Additional file 1

#### Development of domains

The domains were more often developed by factor analysis (n = 36, 34.6%) than by a priori specifications (n = 16, 15.4%). For half of the instruments, the development of the domains was not reported (n = 42, 40.4%) or was not applicable (n = 8, 7.7%). The domains of two instruments were developed by Rasch analysis.

#### Test-retest

Test-retest reliability was assessed for 63 instruments (60.6%). The most frequently used statistical methods were intraclass correlation coefficients either alone (n = 18) or together with other methods (n = 5). This was followed by Pearson correlation coefficient (n = 10), unspecified correlations (n = 9), various types of t-tests (either alone or together with other methods, n = 6) and various other methods (n = 15). 41 development studies (39.4%) did not report on assessing test-retest reliability.

#### Internal consistency

Internal consistency was assessed in 62 development studies (59.6%). Most frequently internal consistency was assessed by Cronbach's alpha alone (n = 46) or Cronbach's alpha together with other methods (n = 10).

#### Validity

Eighty-eight studies reported on the assessment of validity (84.6%). The most frequently assessed type of validity that the authors described was construct validity (n = 43), followed by convergent/convergence validity (n = 19), discriminant validity (n = 18), concurrent validity (n = 16), content validity (n = 12), criterion validity (n = 11), predictive validity (n = 6), divergent validity (n = 4) and face validity (n = 4). For 25 instruments, the authors did not specify or name the type of validity tested. Most authors reported several types of validity. Validity was most frequently assessed with a correlational approach.

#### Responsiveness

The assessment of responsiveness was reported for 20 instruments only (19.2%). Several methods were used.

#### MID

Only 3 development studies reported on the MID (2.9%).

#### Summary of initial validation assessments according to aim of instrument

Table 3 refers to the instruments for which an aim was clearly described before the instrument was developed (n = 49, some studies described more than one aim). The table shows the number and percentage of instruments which assessed each psychometric property. The majority of instruments with a defined aim assessed validity in the initial validation process, regardless of the kind of aim, whereas test-retest was assessed for fewer instruments. For 40.6% of the instruments with an evaluative aim, responsiveness was assessed and the MID for 6.3%.

**Table 3:** Conducted initial validation assessments according to described aims of instruments^1)^

Described aim of instrument	Test-retest		Internal consistency		Validity		Responsiveness		**MID**^**2)**^	
	**n**	**%**^**3)**^	**n**	**%**^**3)**^	**n**	**%**^**3)**^	**n**	**%**^**3)**^	**n**	**%**^**3)**^

Evaluative (n = 33)	23	69.7%	21	63.6%	32	96.9%	13	39.4%	2	6.1%
Discriminative (n = 26)	15	57.7%	18	69.2%	24	92.3%	6	23.1%	1	3.8%
Planning (n = 13)	11	84.6%	7	53.8%	12	92.3%	3	23.1%	1	7.7%
Predictive (n = 5)	1	20.0%	0	0%	3	60.0%	0	0%	0	0%

^1) ^For data extraction details, please see Additional file 1

^2) ^MID = Minimal important difference

^3) ^% in relation to the corresponding aim

## Discussion

Our systematic review showed that there are many existing PRO instruments measuring various dimensions of physical activity, highlighting the importance of this concept as an outcome measure. The methodological quality of the development process varied considerably across the 104 included instruments. For the majority of the instruments, the aim either was not clearly described or not described at all before the instruments were developed. In addition, patients were often not involved in the item identification process of new instruments, making the adaptation of existing scales, unsystematic literature searches and/or expert input the only sources of item generation. Several instruments used quantitative patient input for item selection, but a surprisingly high number of studies did not describe or report on how items were selected. Also, the quality of the initial validation varied widely between the instruments. Internal consistency and test-retest reliability were assessed more frequently than responsiveness to change. The MID was estimated for only 3 instruments. Some instruments defined an evaluative aim; however, responsiveness was assessed in less than half of these. Many studies assessed construct validity while content validity was assessed for only a minority of the instruments.

Over the last decades, physical activity instruments were traditionally used predominantly in epidemiological research to measure physical activity as a potential determinant of health outcomes [1,2]. This requires that the instruments are able to discriminate between people in order to identify different levels of physical activity that might be associated with different health outcomes. In recent years, there has been growing interest in physical activity as a PRO measure. For example in obesity research, studies examine the effect of interventions on physical activity [118-120]. The use of physical activity instruments as outcome measures has implications for the development and initial validation process of these scales. Since PROs should be able to detect changes over time, their evaluative power is essential. Consequently, development and initial validation studies should go beyond cross-sectional studies and assess responsiveness to change and the MID in prospective follow-up studies [7].

PROs for symptoms, health-related quality of life but also for physical activity have become a prevalent outcome in clinical trials. Over the last ten years many new PROs have been developed and validated and it can be expected that in the near future an increasing number of claims on the effectiveness of drugs will be made based on PROs. As a consequence, both the U.S. Food and Drug Administration (FDA) and the European Medicines Agency (EMA) have developed guidance documents on the requirements for PRO instruments that would allow making drug claims. A key evaluation point for the FDA is the evidence on content validity. Content validity describes the extent of how the instrument measures the concept of interest, which is specific to the population, condition and treatments to be studied. The FDA explicitly asks for patient input for item generation through qualitative research to ensure content validity in the development process of a new instrument [121-123].

Although all of the PRO instruments included in this systematic review were developed before the finalisation of the FDA guidance document in December 2009, it is still surprising that in less than one third of the included studies authors reported on qualitative research for item generation such as patient interviews or focus groups, and a minority declared explicitly to have tested content validity of the newly developed instruments. These findings, along with the fact of poor reporting on item selection methods, indicate that only few physical activity PRO instruments would currently fulfil the FDA and EMA requirements for outcome measures. While the need to establish content validity has been recognised for many years, there has been little pressure to conduct qualitative research as illustrated in our systematic review. This is likely to change; at least in the field of clinical trials as investigators developing new instruments can now follow the FDA and EMA guidance to establish content validity more formally through qualitative research. Existing instruments are in a more difficult position, although they may in retrospect support their relevance to patients through additional qualitative research. For example, one may examine whether the constructs measured by existing instruments align with what patients perceive to be important, or if important aspects are missing.

One strength of this systematic review was the adherence to rigorous systematic review methodology along with the broad search strategy to identify existing physical activity instruments and subscales/domains. We supplemented the systematic database searches by a comprehensive hand search as well as by a PROQolid database search. As we aimed to identify any relevant instruments, we kept the inclusion criteria broad by using the definition for physical activity as described in the "2008 Physical activity guideline for Americans" [3]. Such a broad perspective could also be perceived as a limitation. Although we paid great attention to carefully defining the inclusion criteria, we cannot exclude the possibility of having missed questionnaires. Also, the decision about inclusion or exclusion of the instruments was sometimes ambiguous as for example for instruments assessing specific types of physical activity for chronic illnesses such as multiple sclerosis or chronic pain. In such cases we tried to adopt systematically and scientifically defendable decision criteria for inclusion or exclusion. For multiple sclerosis patients, for example, we did not consider physical activity instruments aiming at impaired hand motor activity but we included those assessing physical activity limitations which are more general and which could also be relevant for other chronic illnesses like "Walking ability" [54] or "Physical functioning" [93]. Another example includes activity limitations due to pain, where we excluded some instruments such as those targeting specialised pain coping activities, but included instruments such as the Activities of Daily Living Scale [71]. We focused solely on publications of the development and initial validation, which to some extent may underestimate the rigour of the overall development process. Undoubtedly some instruments might have had additional validation studies which we have not included in this review. However, we suspect that many instruments were introduced into research and practice rather rapidly without further validation, and, if validations were conducted during the development process, it is likely that the authors would have published these results as part of the development paper.

## Conclusion

Our systematic review showed that there are many existing PRO instruments measuring physical activity in chronically ill and elderly patient populations, highlighting the importance of this concept as an outcome measure. However, the development processes often lacked definitions of the instruments' aims and patient input. If PROs for physical activity are to be used in clinical trials, there needs to be more focus on establishing content validity through patient input, and assessing their evaluative measurement properties.

## Competing interests

Anja Frei, Anders Vetsch, Fabienne Dobbels, Laura Jacobs and Milo Puhan have no competing interests. Katja Rüdell is an employee of Pfizer Ltd and Kate Williams is contracted by Pfizer Ltd. The current work is methodological and provides no competitive advantage or disadvantage. No medical writers were used to support the writing of this manuscript.

## Authors' contributions

MP and KR led the systematic review. MP, KR, AF, FD and LJ developed the conceptual framework and the study protocol. MP, KR and AF coordinated the review. MP, FD, KR and AF conducted the electronic database searches; KW, AV, AF, KR and MP conducted the additional searches. AF coordinated the references in RefWorks. KR and FD (1^st ^reviewers), KW and LJ (2^nd ^reviewers) and MP and AF (3^rd ^reviewers) screened titles and abstracts. KW and KR (1^st ^reviewers), AF and AV (2^nd ^reviewers) and MP (3^rd ^reviewer) assessed full texts of the identified studies and extracted the relevant data. AF conducted the statistical analysis. AF and MP drafted the manuscript. All authors contributed to revising the manuscript and approved the final version.

## Authors' information

Katja Rüdell is an honorary lecturer of health psychology at the University of Kent, UK

## Supplementary Material

Additional file 1 **Data extraction results: Development and initial validation process of the reviewed instruments**. Summary of the extracted data for the development and initial validation process of the reviewed instruments according to the categories aim of instruments, identification of items, selection of items (item reduction), development of domains, test-retest, internal consistency, validity, responsiveness and MID. Click here for file

Additional file 2 **References list of excluded articles after full text assessment**. List of all references of articles which have been excluded after full text assessment. Click here for file
