# Supplementary material for: A comprehensive systematic review of the development process of 104 patient-reported outcomes (PROs) for physical activity in chronically ill and elderly people
Source: Health Qual Life Outcomes. 2011 Dec 20;9:116. doi: 10.1186/1477-7525-9-116 (PMC3311097; doi:10.1186/1477-7525-9-116)
Supplement: Additional file 1 — Data extraction results: Development and initial validation process of the reviewed instruments. Summary of the extracted data for the development and initial validation process of the reviewed instruments according to the categories aim of instruments, identification of items, selection of items (item reduction), development of domains, test-retest, internal consistency, validity, responsiveness and MID. [file 1477-7525-9-116-S1.DOC]

**Additional file 1: Data extraction results: Development and initial validation process of the reviewed instruments**

| **Instrument** | **Study** | **Aim of instrument** | **Identification of items** | **Selection of items (Item reduction)** | **Develop-ment of domains** | **Test-retest** | **Internal consistency** | **Validity** | **Responsiveness** | **Minimally important difference (MID)** |
| --- | --- | --- | --- | --- | --- | --- | --- | --- | --- | --- |
| Questionnaire on Impact of COPD on Activities of Daily Living | Alvarez-Gutierrez et al. 2007 | Not clearly described but presumably discriminative | Adaption of The Confronting COPD International Survey (Rennard et al., 2002) | Not reported | Not reported | Not reported | Not reported | Not reported | Not reported | Not reported |
| The Pain Disability Questionnaire (PDQ) | Anagnostis et al. 2004 | Not described | Adaption of the SF-36, McGill Pain Questionnaire, Roland-Morris, Oswestry, Million VAS, and the Multidimensional Pain Inventory  Experts: not specified | Experts: qualitative (formal feedback from expert panel)  Experts: quantitative (relevance) | Factor analysis | Pearson’s correlation | Cronbach's alpha | "Construct validity" (Pearson’s correlation) | Independent sample t-tests | Not reported |
| Chronic Pain Self-efficacy Scale | Anderson et al. 1995 | Not clearly described but presumably evaluative | Adaption of Arthritis Self-Efficacy Scale (ASE)  Literature: not specified | Patients: qualitative (focus groups) | Factor analysis | Not reported | Cronbach's alpha | "Construct validity" (Pearson’s correlation. Multiple regression analysis) | Not reported | Not reported |
| The Activities Checklist | Arbuckle et al. 1994 | Not clearly described but presumably discriminative | Elderly (open ended survey) | Not reported | Not reported | Correlations | Cronbach's alpha | "Face validity" (t-tests)  "Construct validity" (correlations) | Not reported | Not reported |
| Instrumental Activities of Daily Living (IADL) | Avlund et al. 1993 | Discriminative | Not clearly described, but presumably from literature and experts | Patients: quantitative (Rasch analysis) | Rasch analysis | Not reported | Rasch analysis | "Construct validity" (Rasch item analysis) | Not reported | Not reported |
| Questionnaire of Functional Ability | Avlund et al. 1996 | Discriminative | Literature (including pilot study) | Patients: quantitative (Rasch analysis) | Rasch analysis | Not reported | Rasch analysis | "Construct validity" (Rasch item analysis)  “Content validity” (correlations) | Not reported | Not reported |
| Chronic Urticaria Quality of Life Questionnaire (CU-Q2oL) | Baiardini et al. 2005 | Evaluative | Patients (semi-structured interviews)  Experts (panel) | Patients: quantitative (prevalence, importance)  Experts: qualitative (redundancy, ambiguity, difficult to understand, expressed negatively) | Factor analysis | Inter class coefficients | Cronbach's alpha | "Convergent validity" (Pearson's correlation) | Wilcoxon test | Not reported |
| The Sickness Impact Profile (SIP) | Bergner et al. 1981 | Evaluative, planning, discriminative | Patients (open ended questions asking for statements about health)  (Bergner 1976) | Experts: qualitative (independently grouping items together)  Experts: quantitative (rating against criteria)  (Bergner 1976) | A priori (qualitative grouped by experts)  (Bergner 1976) | Correlations (r) | Cronbach’s alpha | “Convergent and discriminant validity” (multitrait-multimethod technique - correlations)  “clinical validity” | Not reported | Not reported |
| Adapted Falls Efficacy Scale (FES) | Bula et al. 2008 | Not clearly described but presumably discriminative and predictive | Elderly | Not reported | Not reported | Intraclass correlation coefficient | Cronbach's alpha | "Construct validity" (Pearson’s correlation)  "Predictive validity" (Multivariate linear regression analyses) | Not reported | Not reported |
| Fibromyalgia Impact Questionnaire | Burckhardt et al. 1991 | Not described | Patients (clinical interactions)  Literature (unsystematic search) | Not reported | Factor analysis | Pearson correlation | Not reported | “Content validity”  “Construct validity” (correlations, PCA) | Not reported | Not reported |
| Bath Ankylosing Spondylitis Functional Index | Calin et al. 1994 | Not clearly described but presumably evaluative, and planning | Experts (not specified)  Patients (not specified) | Patients: qualitative (ambiguity/clarity) | Not reported | Pearson correlation | Not reported | “External validation” (ANOVA) | Pre- and post-treatment mean score correlations | Not reported |
| The Impact on Participation and Autonomy (IPA) | Cardol et al. 1999 | Not clearly described but presumably evaluative | Literature (International Classification of Impairments, Disabilities and Handicaps (ICIDH) manual)  Experts (not specified)  Patients (qualitative study, not further specified) | Patients: quantitative (relevance) | Factor analysis | Not reported | Cronbach's alpha, item total correlation | "Face validity" (expert review)  "Construct validity" (factor analysis) | Not reported | Not reported |
| The Impact on Participation and Autonomy Questionnaire (IPAQ) | Cardol et al. 2001 | Evaluative and discriminative | Literature (International Classification of Impairments, Disabilities and Handicaps (ICIDH) manual)  Experts (not specified)  Patients (not specified) | Patients: qualitative (not specified) | Factor analysis | Cohen's weighted Kappa  Intraclass correlation coefficient | Cronbach's alpha | "Construct validity" (factor analysis)  "Convergent and discriminant validity" (Pearson’s product moment correlation coefficient) | Not reported | Not reported |
| The “Maugeri” Foundation” Respiratory Failure item set (MRF-28), as subdivided by principial components analysis | Carone et al. 1999 | Discriminative | Literature (unsystematic search)  Patients (interviews)  Experts (interviews) | Patients: qualitative (relevance/understanding)  Experts: qualitative (not reported)  Patients: quantitative (factor analysis) | Factor analysis | Not reported | Not reported | “Validity” (Spearman correlations)  “Convergence Validity” (multitrait multimetric analysis) | Not reported | Not reported |
| Zutphen Physical Activity Questionnaire | Caspersen et al. 1991 | Predictive | Adaptation of questionnaire for retired men (Morris, 1984) | Not reported | Not reported | Not reported | Not reported | Not reported | Not reported | Not reported |
| Hong Kong Chinese Everyday Competence Scale | Chou 2003 | Not described | Not reported | Not reported | A priori | Not reported | Cronbach's alpha | "Concurrent validity" (correlations) | Not reported | Not reported |
| The Adelaide Activities Profile (AAP) | Clark & Bond 1995 | Discriminative | Adaption of the Frenchay Activities Index (FAI)  Additional items (source not clearly described) | Patients: quantitative (interpretation, relevance, factor analysis) | Factor analysis | Not reported | Reliability coefficient theta | "Construct validity" (correlations) | Not reported | Not reported |
| Steadiness Score | Clark et al. 2005 | Not clearly described but presumably predictive | Literature (not specified) | Not reported | Not reported | Not reported | Cronbach's alpha | "Construct validity" (Pearson correlation)  "Predictive validity" (regression) | Not reported | Not reported |
| The Customary Activity Questionnaire | Dallosso et al. 1988 | Not clearly described, but presumably discriminative | Not reported | Not reported | Not reported | Correlations (type not specified) | Not reported | “Validity” (correlations) | Not reported | Not reported |
| Exercise Self-Regulatory Efficacy Scale (Ex-SRES) | Davis et al. 2007 | Not clearly described but presumable evaluative | Patient (one-on-one interviews)  Literature (unsystematic search) | Not reported | A priori | Not reported | Cronbach’s Alpha, item-to-total correlation | ”Validity” (correlations) | Not reported | Not reported |
| The YALE Physical Activity Survey for Older Adults (YPAS) | Dipietro et al. 1993 | Not clearly described, but presumably discriminative | Not reported | Not reported | Not reported | Paired t-test  Pearson product moment correlation coefficient | Not reported | “Validity” (Spearman ranked correlation coefficient) | Not reported | Not reported |
| Adapted Barthel Index | Dorevitch et al. 1992 | Not described | Adaptation of Barthel Index | Not reported | Not reported | Not reported | Not applicable | “Concurrent validity” (Wilcoxon signed rank test/ median differences) | Not reported | Not reported |
| Chronic Heart Failure Assessment Tool (CHAT) | Dunderdale et al. 2008 | Not clearly described, but presumably evaluative | Patients (interviews) | Patients: qualitative (acceptability / practicability)  Patients: quantitative (factor analysis) | Factor analysis | Not reported | Cronbach's Alpha, total items correlation | “Criterion validity” (correlations) | Not reported | Not reported |
| University of California San Diego Shortness of Breath Questionnaire (SOBQ) | Eakin et al. 1998 | Evaluative, planning | Not reported in this article | Patients: quantitative (missing data analyses) | A priori | Not reported | Cronbach’s alpha, corrected item total correlation | Not specified which type of validity (correlation with other variables - exercise tolerance, lung function, QOL and depression) | Not reported | Not reported |
| Meaningful Activities of Participation Assessment (MAPA) | Eakman 2007 | Not described | Literature (unsystematic)  Adaptation of the items of the Everyday Activity Checklist (Arbuckle et al, 1994)  Patients | Patients: qualitative (focus group)  Experts: qualitative (guides discussion) | A priori | Pearson's correlation | Cronbach's Alpha | “Construct validity” (by 6 main hypothesis + 2 additional approaches: 1. Cluster analysis, 2. Causal model..)  “Criterion validity' | Not reported | Not reported |
| The OARS Multidimensional Functional Assessment Questionnaire (OMFAQ) | Fillenbaum & Smyer 1981 | Discriminative, evaluative, planning | Adaptation of the OARS Clinical Instrument and the OARS Community Survey Questionnaire (CSQ) | Patients: quantitative (discriminatory power)  Experts: qualitative (not reported) | Not reported | Tested (method not specified) | Not reported | “Validity” (Spearman rank order correlation)  “Content validity”  “Consensual validity”  “Criterion Validity” (Kendall’s tau and Spearman’s rank order correlations) | Not reported | Not reported |
| The Five Item Instrumental Activities of Daily Living Scale | Fillenbaum 1985 | Discriminative, predictive | Adaptation of the ORAS Multidimensional Functional Assessment Questionnaire IADL scale (OMAFQ) | Patients: quantitative (factor analysis) | N/A | Not reported | Not reported | "Discriminant validity"  "Predictive validity" | Not reported | Not reported |
| The magnitude estimation scale | Finch et al. 1995 | Not clearly described, but presumably evaluative, discriminative and planning | Adaptation of the basic set of ADLs purposed by Katz (1963)  Literature (unsystematic search ["several IADL measures"]) | Experts quantitative (relevance, importance, weights) | A priori | Pearson product-moment correlation (of the different approaches to measuring ADL dependencies) | Not reported | "Face validity" (expert appraisal) | Not reported | Not reported |
| Daily Activity Diary for Chronic Pain Patients | Follick et al. 1984 | Evaluative | Not reported | Not reported | Not reported | Pearson correlation coefficients | Not reported | "Validity" (Pearson correlation coefficient) | t-tests, comparing the means for each category | Not reported |
| Household Activities - Activities of Daily Living Scale (HHA-ADL) | Frederiks et al. 1990 | Not clearly described but presumably discriminative and planning | Elderly people | Not reported | Unclear | Cohen's Kappa (questionnaire vs. oral interview) | Cronbach's Alpha | “Discriminant validity”  “Face validity” | Not reported | Not reported |
| The Lifetime Total Physical Activity Questionnaire | Friedenreich et al. 1998 | Not clearly described, but presumably predictive | Not clearly described, presumably based on adaptation of questionnaires and experts (input from the authors) | Patients: qualitative (cognitive interviews) | A priori | Test-retest correlations  T-tests | Not reported | Not reported | Not reported | Not reported |
| Health Assessment Questionnaire | Fries et al. 1982 | Not described | Literature (not reported)  Patients (interviews)  Experts (interviews) | Patients: quantitative (factor analysis) | Not reported | Correlations (not specified) | Correlations | “Validity” (correlations) | Not reported | Not reported |
| Population surveys of chronic disease and disability | Garrad 1971 | Not clearly described, but presumably discriminative | Experts (not clearly described)  Patients | Patients: quantitative (factor analysis) | A priori | Tested (method not specified) | Not reported | "Validity" (comparison with clinical assessments) | Not reported | Not reported |
| London Chest Activity of Daily Living Scale (LCADL) | Garrod et al. 2000 | Not clearly described, but presumably evaluative and discriminative | Patients (interviews)  Literature (unsystematic search) | Patients: quantitative (poor discriminative ability, correlations with age/gender, poor test-retest reliability, factor analysis) | Factor analysis | Not assessed  (Kendall tau rank correlation coefficient used for item reduction, previous 17-item-scale) | Cronbach's alpha | "Construct validity" (comparison of mean scores)  "Concurrent validity" (Spearmans's Rho correlations, ANOVA) | Not reported | Not reported |
| The Chronic Respiratory Disease Questionnaire (CRQ) | Guyatt et al. 1987 | Not clearly described, but presumably evaluative and discriminative | Literature (unsystematic search)  Experts (not specified)  Patients (interview) | Patients: quantitative (importance x frequency weighting) | Not reported | Coefficient of variation | Not reported | "Validity" (correlations) | Mean changes (t-tests) after administering interventions where changes were expected  t values (comparison with t values of other instruments) | Not reported |
| The Geriatric Quality of Life Questionnaire (GQLQ) | Guyatt et al. 1993 | Evaluative | Literature (unsystematic search)  Experts (interview) | Patients: qualitative (interviews)  Patients: qualitative (importance) | Not reported | Not reported | Not reported | "Validity" (Pearson’s correlations) | Responsiveness coefficient (= Guyatt coefficient)  ratio: differences in the subjects who changed divided by the standard deviation of the difference between scores in adjacent visits in stable patients | 80 per group (for the ADL domain) |
| London Handicap Scale | Harwood et al. 1994 | Evaluative | Adaptation of the International Classification of Impairments, Disabilities and Handicaps  Patients (interviews)  Experts (interviews) | Not reported | Not reported | Not reported | Not reported | “Validity” (Pearson correlation coefficient, Kendall’s coefficient [tau]) | Not reported | Not reported |
| Osteoporosis Functional Disability Questionnaire | Helmes et al. 1995 | Evaluative | Literature (not reported)  Patients (interviews)  Experts (interviews) | Not reported | Not reported | Pearson correlation | Cronbach’s alpha | “Criterion validity” (correlations)  “Construct validity” (correlations) | Chi-square, t-test, Pearson correlations | Not reported |
| The Duke Activity Status Index (DASI) | Hlatky et al. 1989 | Not clearly described, but presumably evaluative and predictive | Literature (unsystematic search) | Patients: qualitative (interview, ability to perform activities)  Patients: quantitative (exploratory analysis) | N/A | Not reported | Not reported | “Validity” (Spearman correlation [with objective tests]) | Not reported | Not reported |
| Multiple Sclerosis Walking Scale (MSWS-12) | Hobart et al. 2003 | Not clearly described but presumably discriminative and evaluative. | Patients (interviews)  Experts (interviews)  Literature review (not reported) | Not reported | Not reported | Intraclass correlation coefficient | Cronbach’s alpha | “Convergent validity” (correlations)  “Discriminant validity » | Effect size  Standardised response means  Relative responsiveness (relative efficiency) | Not reported |
| Activities Index | Holbrook & Skilbeck 1983 | Not clearly described, but presumably evaluative, discriminative and predictive | Patients (interview) | Patients: quantitative (factor analysis)  Experts: qualitative (relevance) | Factor analysis | Not reported | Not reported | Not reported | Not reported | Not reported |
| The Living with Asthma Questionnaire | Hyland 1991 | Planning, evaluative | Patients (focus groups) | Patients: quantitative (factor analysis) | A priori  Factor analysis (has been done, but there were no statistical reason for identifying subscales appropriate to different domains) | Tested, but method not specified | Not reported | “Convergent validity” (correlations)  “Predictive validity” | Not reported | Not reported |
| Respiratory Illness Questionnaire-monitoring 10 (RIO-MON10) | Jacobs et al. 2004 | Not clearly described, but presumably evaluative, discriminative and planning | Adaptation of QOL-RIO (Maille et al., 1997) | Patients: quantitative (factor analysis, Cronbach's alpha, interscale distribution)  Experts: quantitative (relevance: suitability of the contents of the short form) | Factor analysis | Intraclass correlation | Cronbach's alpha | "Construct validity" (Spearman correlation)  "Criterion validity" (Spearman correlation) | Discriminative responsiveness:  Wilcoxon test: mean difference in scores between subgroups of highest and lowest quarter, between mean short form scores and subgroup scores of reported dyspnoea  Responsiveness to change  Standardised response mean (SRM): mean change in score divided by the standard deviation of the change in score | 0.5 |
| Functional Status Assessment Instrument | Jette & Deniston 1978 | Planning, evaluative | Adaptation from Katz | Expert: quantitative (relevance) | Factor analysis | Agreement ratios by interviewers  Intraclass correlation coefficient (ICC) | Not reported | “Convergent validity” | Not reported | Not reported |
| The St. George's Respiratory Questionnaire (SGRQ) | Jones et al. 1992 | Not clearly described, but presumably evaluative and discriminative | Not reported | Not reported | Principal component analysis | Intraclass correlation, coefficient of variation | Not reported | “Convergence validity” (coefficient of correlation (r2-values), ANOVA) | Correlations, ANOVA | Not reported |
| The Quality of Well-Being Scale, Version 1.04 (QWB) | Kaplan et al. 1997 | Not described | Literature (reviews, not further specified)  (Kaplan 1998) | Not reported | Not reported | Correlations (not specified) | Cohen’s alpha  (Kaplan 1998) | Not reported | Not reported | Not reported |
| The Functional Independence Measure (FIM) | Keith et al. 1987 | Not clearly described but presumably evaluative, planning, predictive and discriminative | Literature (unsystematic search) | Experts: qualitative (clarity etc) | Not reported | Not reported | Not reported | Not reported | Not reported | Not reported |
| Hierarchial Polychotomous ADL-IADL Scale (developed further to Groningen Activity Restriction Scale (GARS) (Kempen et al., 1996) | Kempen & Suurmeijer 1990 | Not clearly described, but presumably discriminative, predictive and planning | Patients (interview) | Experts: quantitative (relevance) | Factor analysis (Principal component analysis) | Not reported | Cronbach’s alpha | ”Validity” (Pearson correlation) | Not reported | Not reported |
| COPD-Disability-Index (CDI) | Kühl 2009 | Discriminative | Adaptation of Pain-Disability-Indes (PDI) | Not reported | A priori  factor analysis | Not reported | Cronbach's alpha | "Convergent validity" (Pearson correlations)  "Divergent validity" (Pearson correlations) | Not reported | Not reported |
| The Pulmonary Functional Status and Dypnea Questionnaire (PFSDQ) | Lareau et al. 1994 | Evaluative, discriminative | Patients (interviews)  Experts (reviewed items for content) | Experts: qualitative (reviewed items for content) | Not reported | Not reported | Cronbach's alpha | “Content validity” (experts’ examination)  “Concurrent validity” (comparison of scores)  "Construct validity" (student’s t-test) | Not reported | Not reported |
| The modified version of the Pulmonary Functional Status and Dyspnea Questionnaire (PFSDQ-M) | Lareau et al. 1998 | Evaluative | Adaptation of The Pulmonary Functional Status and Dyspnea Questionnaire (PFSDQ) | Patients: quantitative (items with response rate <80%, floor/ceiling effect, item-to-item correlations) | Factor analysis | Correlation coefficient | Cronbach's alpha  inter-item correlations | “Construct validity” – by examination of "convergent validity" (principal component extraction, varimax rotation)  "Divergent validity" (principal component extraction, varimax rotation) | "Differences in total scores between groups based on rate of loss of lung function" | Not reported |
| Dyspnea Questionnaire | Lee et al. 1998 | Not clearly described, but presumably evaluative, discriminative and planning | Literature (unsystematic search)  Experts (not clearly described) | Patients: qualitative (comprehension/ informativeness) | Not reported | Not reported | Cronbach’s alpha  Inter-item correlations  Factor analysis | Not reported | Not reported | Not reported |
| The Functional Performance Inventory (FPI) | Leidy 1999 | Discriminative, evaluative | Literature (unsystematic search)  Patients (interview, questionnaire: relevance, clarity, comprehensiveness) | Experts: qualitative (rating of relevance, content validity index)  Patients: qualitative (interviews) | A priori, factor analysis | Intraclass correlation | Cronbach's alpha | "Concurrent validity" (Pearson correlation coefficient)  "Construct validity" (Pearson correlation coefficient)  "Discriminant validity" (Independent-Samples t-test) | Not reported | Not reported |
| Work Limitations Questionnaire | Lerner et al. 2001 | Not clearly described but presumably evaluative | Patients (focus groups) | Patients: qualitative (cognitive interviews)  Patients: quantitative (alternate forms test) | Factor analysis | Not reported | Cronbach’s alpha | “Construct validity”  “Relative validity” (multiple linear regression) | Not reported | Not reported |
| The Asthma Impact Record (AIR) Index | Letrait et al. 1996 | Not clearly described, but presumably evaluative | Patients (interviews) | Patients: quantitative (factor analysis)  Other methods (not specified) | Factor analysis | Intraclass correlation coefficients | Cronbach’s alpha | “Concurrent validity” (Spearmans rank correlations, Wilcoxon’s and Kruskal-Wallis tests) | Not reported | Not reported |
| The Cardiovascular Limitations and Symptoms Profile (CLASP) | Lewin et al. 2002 | Not clearly described, but presumably discriminative and evaluative | Not reported | Not reported | Factor analysis | Pearson’s r correlations | Cronbach’s alpha | “Validity” (Pearson’s correlation coefficients) | Not reported | Not reported |
| Activities of Daily Living Scale | Linton 1990 | Discriminative, evaluative | Not reported | Not reported | Not reported | “Test-retest reliability” (method not reported) | Assessed but method not reported | "Validity" (Pearson correlation) | Not reported | Not reported |
| The Physical Activity Questionnaire | Liu et al. 2001 | Not described | Adaptation of YPAS (Di Pietro et al., 1993) and other questionnaires (Taylor, 1978; Capersen et al., 1991; McArdle et al., 1991)  Patients (focus groups) | Patients: qualitative (focus group; adaptation to the Hong Kong Chinese population) | A priori | Intraclass correlation (ICC) | Not reported | “Validity” (t-test, Bland and Altman plot: comparing TEE (daily energy expenditure) values, from questionnaire and from calculated values using measured BMR (basal metabolic rate) multiplying by PAL (physical activity level) | Not reported | Not reported |
| The Quality of Life Respiratory Illness Questionnaire (QOL-RIQ) | Maillé et al. 1997 | Discriminative | Literature (unsystematic search)  Experts (interview) | Patients: qualitative (interview)  Patients: quantitative (factor analysis) | Factor analysis | Not reported | Cronbach’s alpha  Split-half reliabilities | “Construct validity” (Spearman-rank coefficients) | Not reported | Not reported |
| The Leisure Time Physical Activity Instrument (LTPAI) | Mannerkorpi & Hernelid 2005 | Not clearly described, but presumably discriminative | Literature (unsystematic search) | Patients: qualitative (relevance)  Experts: qualitative (not specified) | Not reported | Intraclass correlation coefficient, Kappa statistics, Wilcoxon's signed rank, spearman's correlation coefficient | Not reported | "Construct validity" (Spearman's correlation coefficients) | Not reported | Not reported |
| The Physical Activity at Home or at Work Instrument (PAHWI) | Mannerkorpi & Hernelid 2005 | Not clearly described, but presumably discriminative | Literature (unsystematic search) | Patients: qualitative (relevance)  Experts: qualitative | Not reported | Intraclass correlation coefficients, Wilcoxon's signed rank test | Not reported | Not reported | Not reported | Not reported |
| The Immune Thrombocytopenic Purpura Patient Assessment Questionnaire (ITP-PAQ) | Mathias et al. 2007 | Evaluative | Literature (unsystematic search)  Patients (focus group of patients with ITP)  Experts (clinical opinion) | Not reported | Factor analysis | Intra-class correlation coefficients Pearson correlation coefficient | Cronbach's alpha | "Convergent construct validity" (Pearson's correlation coefficients)  "Discriminant construct validity" (Pearson's correlation coefficients)  "Known groups validity" (t statistic) | Guyatt's statistic  1-sample t-test  standardized response mean (SRM)  effect size (ES) | Not reported |
| Scale for the Instrumental Activities of Daily Living in the Elderly (IADL-EDR) | Mathuranath et al. 2005 | Discriminative | Adaptation of IADL scale of Lawton & Brody (1969) | Experts, elderly, self-help group, and significant others: qualitative (consultation)  Patients: quantitative (appropriateness of gradation of response, low rate of applicability) | A priori | Kendall tau rank correlation coefficient | Cronbach's alpha | "Criterion validity" (ROC curve, detection rate)  "Construct validity" (Kendall tau rank correlation coefficient) | Score difference between 2 rounds of testing, correlations | Not reported |
| The Multidimensional Task Ability Profile (MTAP) | Mayer et al. 2005 | Discriminative, planning | Literature review (unsystematic) The MTAP is based on items from the SFS (Spinal Function Sort) and the HFS (Hand Function Sort)  Experts (not specified) | Method not specified | Not reported | Intraclass correlation coefficients  Pearson product moment correlations | Split-half procedure (intraclass correlation coefficients) | “Concurrent validity” (Spearman correlations) | Not reported | Not reported |
| The MOS 36-Item Short-Form Survey (SF-36) | McHorney et al. 1994 | Not reported (in McHorney et al. 1994)  Reported in Ware et al. 1992 :  Evaluative, discriminative | Not reported in McHorney et al. 1994.  Information from Ware et al. 1992:  Literature (unsystematic search)  Adaptation of the full-length MOS scale  Experts (using the corresponding full-length MOS scale as criterion in selecting items for the SF-36) | Not reported in McHorney et al.  Information from Ware et al. 1992:  Patients: quantitative (factor analysis) | Not reported in McHorney et al.  Information from Ware et al. 1992:  A priori | Not reported | Cronbach’s alpha coefficients | “Discriminant validity” (Scaling success rates were computed for each scale as the percentage of item scaling successes (testes passed) relative to the total number of item scaling tests) | Not reported | Not reported |
| The Dyspnea Management Questionnaire (DMQ) | Migliore et al. 2006 | Planning, evaluative | Significant others (qualitative interviews in a previous study)  Literature (unsystematic search)  Adaptation of "The COPD Self-Efficacy scale" for the self efficacy for activity subscale | Patients: quantitative (factor analysis)  Experts: quantitative (relevance) | A priori | Intra-class correlation coefficients | Cronbach's alpha  (Pearson-) correlations between item scores  corrected item-total scores | "Concurrent validity" (Pearson correlations)  "Discriminant validity" (Pearson correlations)  "Divergent construct validity, criterion-related validity" (method not described) | Not reported | Not reported |
| The Centers for Disease Control and Prevention’s Healthy Days Measures (the CDC HRQOL-14) | Moriarty et al. 2003  Atlanta Georgia CDC 2000 [http://www.cdc.gov/hrqol/] | Not clearly described, but presumably discriminative, evaluative and planning | Experts (two workshops [1991 and 1992] not further specified) | Experts (two workshops [1991 and 1992] not further specified) | Not reported | Not reported | Not reported | Not reported | Not reported | Not reported |
| The Chronic Obstructive Pulmonary Disease Activity Rating Scale (CARS) | Morimoto et al. 2003 | Not clearly described, but presumably discriminative | Literature (unsystematic search, containing "ADL" by Katz et al. 1963, "IADL" by Fillenbaum 1985, and "social activity" by Lawton & Brody 1969) | Patients: quantitative (frequency distribution / corrected item total correlation) | Factor analysis | Pearson correlation coefficients (unsure) | Cronbach's alpha | "Concurrent validity" (Pearson correlation coefficients)  "Construct Validity" | Relationship of the score distribution between total CARS score and degree of limitation of daily life (4 classes) | Not reported |
| The IOWA Self-Assessment Inventory | Morris et al. 1989 | Not described | Not reported | Not reported | Not reported | Not reported | Cronbach's alpha | "Construct validity" | Not reported | Not reported |
| The Veterans Specific Activity Questionnaire (VSAQ) | Myers et al. 1994 | Predictive | Experts (not specified, info directly from the author) | Experts (not specified, info directly from the author) | N/A | Not reported | Not reported | Not reported | Not reported | Not reported |
| The Chronic Fatigue Syndrome-Activities and Participation Questionnaire (CFS-APQ) (Dutch version assessed) | Nijs et al. 2003 | Evaluative, planning | Not clearly reported (presumably adaptation of questionnaires; link to WHO ICF homepage) | Not reported | A priori | Intraclass correlation coefficients (ICC) | Cronbach's alpha | "Content validity" (corresponding frequency)  "Convergent validity" (Spearman rank correlation with VAS) | Not reported | Not reported |
| Daily Living Activities Questionnaire | Nouri & Lincoln 1987 | Not clearly described but presumably evaluative and discriminative | Not reported | Not reported | Not reported | Kappa coefficient | Not reported | Not reported | Not reported | Not reported |
| The Duke-UNC Health Profile (DUHP) | Parkerson et al. 1981 | Evaluative | Literature (unsystematic search)  Adaption of Sickness Impact Profile, McMaster Health Index and OARS | Not reported | Not reported | Guttmann coefficient | Cronbach’s alpha | “Content validity”  “Construct validity – convergent and discriminant” (Spearman’s correlation) | Not reported | Not reported |
| The harmonized four-item ADL measure | Pluijm et al. 2005 | Not clearly described, but presumably discriminative | Patients (interview) | Patients quantitative (factor analysis) | Not reported | Not reported | Cronbach's alpha | "Construct validity" (Chi-square, logistic regression)  "Convergent validity" (Chi-square, logistic regression) | Not reported | Not reported |
| Specific Activity Questionaire (SAQ) | Rankin et al. 1996 | Not clearly described, but presumably discriminative | Not reported | Not reported | N/A | Not reported | Not reported | Not reported | Not reported | Not reported |
| The Peripheral Arterial Disease (PAD) Walking Impairment Questionnaire | Regensteiner et al. 1990 | Discriminative, evaluative | Not reported | Not reported | Not reported | Paired t-test | Not reported | “Validity” (Pearson’s product moment correlation coefficient) | Correlation between the change in summary score (for both, speed and distance) between patients before and after treatment | Not reported |
| Fast Functional Performance Inventory | Rejeski et al. 1995 | Not clearly described but presumably evaluative | Literature (unsystematic search)  Patients (focus groups) | Experts: qualitative (panel) | Factor analysis | Reported but unclear which coefficient used | Cronbach’s alpha | “Concurrent and convergent validity” (unclear) | Not reported | Not reported |
| The Self-Efficacy for Exercise Scale (SEE) | Resnick & Jenkins 2000 | Not clearly described, but presumably discriminative | Adaptation of Self-Efficacy barriers to exercise measure (McAuley, 1990, unpublished)  Patients (interviews) | Patients qualitative (rating) | Not reported | Structured equations approach (squared multiple correlation coefficient) | Cronbach's alpha | "Construct validity"  “Criterion related validity” (was tested using concurrent validity”)  Alternative approach to validity testing: "structural equotation modeling" | Not reported | Not reported |
| The Physical Activity and Disability Survey (PADS) | Rimmer et al. 2001 | Not clearly described, presumably evaluative and discriminative | Patients (interview)  Experts (interview)  Literature search (not clear, if systematic or unsystematic) | Patients: quantitative (factor analysis)  Patients: qualitative (rating) | Factor analysis | Intra-class correlations (to assess both, interrater and test-retest reliability) | Cronbach's alpha | "Concurrent validity" (correlational approach)  "Predictive validity" (for both, concurrent and predictive validity tests: correlational approach by comparing the PADS subscale scores with other fitness measures) | Not reported | Not reported |
| Disability Questionnaire | Roland & Morris 1983 | Evaluative, predictive | Adaption of Sickness Impact Profile | Patients: qualitative (relevance) | Not reported | Tested (but correlation coefficient is not specified) | Not reported | “Validity” (unclear) | Not reported | Not reported |
| The RAYS scale | Rotstein et al. 2000 | Evaluative | Experts (interview)  literature (unsystematic) | Experts: quantitative (rating) | Not reported | Not reported | Cronbach's alpha  Item-total correlation  two-sample t-test  Pearson correlation coefficients | "Construct validity" (unpaired t-test)  "Discriminate validity" (non parametric analysis for comparing the median values) | Not reported | Not reported |
| The Cancer Rehabilitation Evaluation System (CARES) | Schag et al. 1990 | Not clearly described, but presumably discriminative and planning | Literature (search type not specified)  Patients (interviews)  Experts (discussions) | Patients: quantitative (factor analysis) | Factor analysis | Percentage agreement / test-retest correlations | Not reported | “Validity” (Pearson product-moment correlations) | Not reported | Not reported |
| Questionnaire of functional ability | Schultz-Larsen et al. 1992 | Discriminative, predictive | Not reported | Patients: quantitative (factor analysis) | Factor analysis | Not reported | Not reported | “Criterion related validity” | Not reported | Not reported |
| Modified Barthel Index | Shah et al. 1989 | Discriminative | Patients (not reported) Adaption of Barthel Index | Not reported | Not reported | Mean correlation | Cronbach’s alpha | Not reported | Not reported | Not reported |
| The 15-D Measure of HRQOL | Sintonen 1994 & 2001 | Evaluative, planning, discriminative | Literature (12D from Sintonen 1981a, 1981b and others, [official Finnish health documents, not further reported]) | Experts: qualitative (not reported)  Patients: qualitative (“feedback”, not further not reported)  Patients: quantitative (factor analysis) | Factor analysis | Repeatability coefficient | Not reported | “Content validity”  “Criterion validity” (correlations)  “Construct validity”, containing convergent and discriminant validity tests (Pearson correlations) | Comparing percentages at the ‘ceiling’ and ‘floor’ and skewness coefficients in two patient groups at baseline and after treatment  Effect size  Standardised mean response (SMR) | 0.02-0.03 |
| Activities of Daily Living Inventory (ADLI) | So & Man 2008 | Evaluative | Adaptation of Chronic Respiratory Questionnaire (CRQ), Modified Barthel Index and Lawton IADL | Patients: qualitative (panel review) | Factor analysis | Not reported | Cronbachs' Alpha  Mean inter-item correlation  Item-total correlation | “Content validity” “Construct validity” (explorative factor analysis) | Not reported | Not reported |
| The LSA Physical Activity Questionnaire (LAPAQ) | Stel et al. 2004 | Not clearly described, but presumably discriminative | Adaptation of the Modified Baecke Questionnaire for Older Adults and the Zutphen Physical Activity Questionnaire | Not reported | N/A | 1-year test-retest: Weighted kappa (total number of activities)  Kappa (each activity [yes/no]) | Not reported | “Construct validity” (Spearman correlation coefficients) | Not reported | Not reported |
| CHAMPS Physical Activity Questionnaire | Stewart et al. 2001 | Evaluative | Literature (unsystematic search) | Not reported | Not reported | Intraclass correlation coefficient | Not reported | “Construct validity” (Pearson’s correlation)  “Known-groups validity” (F-statistics, ANOVA) | Using ANOVA, the extent to which 1-yr changes in physical activity differed between the intervention and control group, controlling or age and gender  Effect size | Not reported |
| Falls Efficacy Scale (FES) | Tinetti et al. 1990 | Not clearly described but presumably discriminative and predictive | Experts (open ended questions) | Experts: qualitative (agreement with previous group of experts)  Patients: quantitative (relevance) | Not reported | Pearson's correlation coefficient | Not reported | Not reported | Not reported | Not reported |
| The Rapid Assessment of Physical Activity (RAPA) | Topolski et al., 2006 | Evaluative, discriminative and planning | Literature (systematic search)  Adaptation of Centers for Disease Control and Prevention (CDC) guidelines | Experts: qualitative (panel)  Patients (older adults): qualitative (focus groups and cognitive debriefing) | A priori | Not reported | Not reported | “Discriminant validity” (known groups analysis, t-tests)  “Criterion validity” (spearman correlation, t-test)  “Predictive validity” (categorisation and comparison with other instrument) | Not reported | Not reported |
| The Seattle Obstructive Lung Disease Questionnaire (SOLQ) | Tu et al. 1997 | Evaluative and presumably discriminative | Patients (interview)  Experts (not exactly described)  Literature (unclear, if systematic or not) | Patients: qualitative (interviews assessed the readability of the questions, the format and ease of self-administration) | Not reported | Intra-class correlation coefficients | Cronbach's alpha | "Construct validity" (correlation with other questionnaires and objective tests) | Guyatt coefficient  minimal clinical important difference is calculated  paired t-test | Not reported |
| The McMaster Toronto Arthritis (MACTAR) Patient Preference Disability Questionnaire | Tugwell et al. 1987 | Evaluative | Not reported | Not reported | Not reported | Not reported | Not reported | Not reported | Not reported | Not reported |
| The Clinical COPD Questionnaire (CCQ) | van der Molen et al. 2003 | Evaluative  discriminative | Patients (interview, focus group)  Experts (item generation by discussions between experts, consultations)  Literature: (unsystematic search: COPD Questionnaires, treatment goals from international guidelines) | Experts: quantitative (relevance; ranking 5-point Likert type scale from extremely important to useless) | A priori | Intra-class correlation coefficients | Cronbach's alpha | "Discriminant validity": (Mann-Whitney-U test)  "Convergent validity": (Spearman's rank correlations)  "Divergent validity": (Spearman's rank correlations) | Wilcoxon U test | Not reported |
| Questionnaire for Physical Activity Decline in pain (PAD) | Verbunt 2008 | Not clearly described, but presumably evaluative | Adaptation of Physical Activity Rating Scale (PARS) Vercoulen 1997 | N/A | N/A | Intraclass coefficient score | Cronbach’s alpha | “Construct validity” (Pearson correlation coefficient)  “Convergent validity” (change in physical activity level)  “Discriminant validity” (level of physical activity) | Not reported | Not reported |
| Physical activity questionnaire | Voorrips et al. 1991 | Discriminative | Adaptation of activity questionnaire (Baecke et al., 1982) | Patients: qualitative (focus group) | Not reported | Student’s t-test  Spearman’s correlation coefficient  Kendall’s tau-b correlation coefficients | Not reported | “Relative validity” (Spearman’s rank correlation, Kendall’s tau-b correlation coefficients) | Not reported | Not reported |
| The Physical Activity Scale for the Elderly (PASE) | Washburn et al. 1993 | Not clearly described, but presumably predictive | Literature (unsystematic)  Experts (interview) | Experts (relevance)  Patients: quantitative (factor analysis)  Patients qualitative (interview) | N/A | Test-retest reliability coefficient | Cronbach’s alpha | “Construct validity” (Pearson correlations [correlation with objective measures]) | Not reported | Not reported |
| The Pulmonary Functional Status Scale (35-item PFSS) | Weaver et al. 1998 | Evaluative | Literature (unsystematic search)  Experts (by modifying items from widely-used instruments) | Patients: quantitative (factor analysis) | Factor analysis | Test-retest correlation coefficient (Spearman-correlation) | Cronbach’s alpha | “Content validity” (panel of experts)  “Construct validity” (exploratory factor analysis)  “Concurrent validity” (Pearson correlation with other scales and objective tests) | Not reported | Not reported |
| The COPD Self-Efficacy Scale (CSES) | Wigal et al. 1991 | Planning, discriminative | Not reported | Patients: quantitative (factor analysis) | Factor analysis | Pearson product-moment correlations  Student’s t-test | Cronbach’s alpha | Not reported | Not reported | Not reported |
| The Severe Respiratory Insufficiency Questionnaire (SRIQ) | Windisch et al. 2003 | Not clearly described but presumably evaluative and discriminative | Patients (interviews) | Experts: qualitative (panel)  Patients: quantitative (item scale correlations) | Factor analysis  (a priori hypothesis could not be approved) | Not reported | Cronbach’s alpha | “Construct validity” (factor analysis)  “Concurrent validity” (Spearman correlation) | Not reported | Not reported |
| The Manchester Respiratory Activities of Daily Living Questionnaire (MRADL) | Yohannes et al. 2000 | Planning, discriminative, evaluative | Adaptation of the Nottingham Extended ADL Questionnaire (NEDAL) and the Breathing Problems Questionnaire (BPQ) | Patients: quantitative (factor analysis) | Not reported | Wilkinson Signed Rank Test | Cronbach’s alpha | “Construct Validity” (relationship with other objective and subjective tests)  “Internal validity” | Significant t-test between pulmonary rehabilitation patients  Pre and post mean scores | Not reported |
| The Activity of Daily Living Dyspnoea scale (ADL-D scale) | Yoza et al. 2009 | Evaluative | Patients (interviews)  Literature (unsystematic search) | Patients: quantitative (relevance)  Experts: quantitative (relevance) | N/A | Not reported | Cronbach’s alpha | “Concurrent validity” (Spearman rank correlation coefficients, Whitney’s U-test)  “Discriminant validity” (Spearman rank correlation coefficients, Whitney’s U-test) | Not reported | Not reported |
| The Quality of Life Questionnaire for Patients With Chronic Respiratory Disease (CV-PERC) | Zaragoza & Lugli-Rivero 2009 | Not clearly described, but presumably discriminative | Adaptation of the St. George Respiratory Questionnaire (SGRQ) and the SF-36 – and 3 new domains from  Patients (interviews)  Literature (unsystematic review) | Experts: quantitative (relevance)  Patients: quantitative (factor analysis) | Factor analysis | Not reported | Cronbach’s alpha | “Construct validity” (Kaiser-meyer-Olkin, Barlett test of sphericity, exploratory factor analysis, principal components analysis with varimax rotation)  “Convergent validity” (correlations between scales)  “Discriminant validity” (correlations between scales)  “Content validity” | Not reported | Not reported |
| Quality of Life Instrument (QOL-Instrument) | Zhou et al. 2009 | Not described | Patients (focus groups)  Experts (not reported)  Literature search (systematic) | Patients: quantitative (factor analysis)  Patients: qualitative (interviews) | A priori and factor analysis | Intraclass-correlation-coefficients (ICC) | Cronbach’s alpha | “Content validity”  “Concurrent validity” (Pearson product-moment correlation)  “Discriminant validity” (t-test, two-tailed P-values)  “construction validity” | Not reported | Not reported |
| Scale of Older Adults Routine (SOAR | Zisberg 2005 | Planning | Literature (unsystematic)  Experts (rating, relevance) | Patients: qualitative (panel review) | A priori | Intraclass correlation coefficients | Due to time being measured traditional measures not possible used | “Content validity” and “Concurrent validity” (Pearson's correlation)  “Construct validity” (Trait routinization)  “Convergent validity” | Not reported | Not reported |
