# Supplementary material for: A comprehensive systematic review of the development process of 104 patient-reported outcomes (PROs) for physical activity in chronically ill and elderly people
Source: Health Qual Life Outcomes. 2011 Dec 20;9:116. doi: 10.1186/1477-7525-9-116 (PMC3311097; doi:10.1186/1477-7525-9-116)
Supplement: Additional file 2 — References list of excluded articles after full text assessment. List of all references of articles which have been excluded after full text assessment. [file 1477-7525-9-116-S2.DOC]

**Additional file 2: References list of excluded articles after full text assessment**

**1.** Ainsworth BE, Jacobs DR, Jr., Leon AS. Validity and reliability of self-reported physical activity status: the Lipid Research Clinics questionnaire. *Medicine and science in sports and exercise.* 1993;25(1):92-98.

**2.** Ainsworth BE, Leon AS, Richardson MT, Jacobs DR, Paffenbarger RS, Jr. Accuracy of the College Alumnus Physical Activity Questionnaire. *Journal of clinical epidemiology.* 1993;46(12):1403-1411.

**3.** Almeida MH, de Pinho Spinola AW, Iwamizu PS, Okura RI, Barroso LP, Lima AC. [Reliability of the instrument for classifying elderly people's capacity for self-care]. *Revista de saude publica.* 2008;42(2):317-323.

**4.** Anders J, Dapp U, Laub S, von RentelnKruse W. Impact of fall risk and fear of falling on mobility of independently living senior citizens transitioning to frailty: Screening results concerning fall prevention in the community. *Zeitschrift fur Gerontologie und Geriatrie.* 2007;40(4):255-267.

**5.** Araki A, Izumo Y, Inoue J, et al. [Development of Elderly Diabetes Impact Scales (EDIS) in elderly patients with diabetes mellitus]. *Nippon Ronen Igakkai Zasshi - Japanese Journal of Geriatrics.* 1995;32(12):786-796.

**6.** Arbuckle TY, Gold D, Andres D. Cognitive functioning of older people in relation to social and personality variables. *Psychology and aging.* 1986;1(1):55-62.

**7.** Avlund K, Kreiner S, Schultz-Larsen K. Construct validation and the Rasch model: functional ability of healthy elderly people. *Scandinavian journal of social medicine.* 1993;21(4):233-246.

**8.** Badia X, Webb SM, Prieto L, Lara N. Acromegaly Quality of Life Questionnaire (AcroQoL). *Health & Quality of Life Outcomes.* 2004;2(Journal Article):13.

**9.** Baecke JA, Burema J, Frijters JE. A short questionnaire for the measurement of habitual physical activity in epidemiological studies. *The American Journal of Clinical Nutrition.* 1982;36(5):936-942.

**10.** Bandura A. Self-efficacy: The exercise of control. *Freeman & Co.* 1997.

**11.** Barber JH, Wallis JB, McKeating E. A postal screening questionnaire in preventive geriatric care. *The Journal of the Royal College of General Practitioners.* 1980;30(210):49-51.

**12.** Barberger-Gateau P, Commenges D, Gagnon M, Letenneur L, Sauvel C, Dartigues J. Instrumental Activities of Daily Living as a screening tool for cognitive impairment and dementia in elderly community dwellers. *Journal of the American Geriatrics Society.* 1992;40(11):1129-1134.

**13.** Barberger-Gateau P, Rainville C, Letenneur L, Dartigues J. A hierarchical model of domains of disablement in the elderly: a longitudinal approach. *Disability & Rehabilitation.* 2000;22(7):308-317.

**14.** Basler HD, Luckmann J, Wolf U, Quint S. Fear-avoidance beliefs, physical activity, and disability in elderly individuals with chronic low back pain and healthy controls. *Clinical Journal of Pain.* 2008;24(7):604-610.

**15.** Bayliss EA, Ellis JL, Steiner JF. Subjective assessments of comorbidity correlate with quality of life health outcomes: initial validation of a comorbidity assessment instrument. *Health & Quality of Life Outcomes.* 2005;3(Journal Article):51.

**16.** Bennell KL, Hinman RS, Crossley KM, et al. Is the Human Activity Profile a useful measure in people with knee osteoarthritis? *Journal of Rehabilitation Research & Development.* 2004;41(4):621-629.

**17.** Berg K W-DSLWJ. Measuring balance in the elderly: preliminary development of an instrument. *Physiother Can.* 1989(Journal Article).

**18.** Bergland A, Jarnlo G, Laake K. Validity of an index of self-reported walking for balance and falls in elderly women. *Advances in Physiotherapy.* 2002;4(2):65-73.

**19.** Bestall JC, Paul EA, Garrod R, Garnham R, Jones PW, Wedzicha JA. Usefulness of the Medical Research Council (MRC) dyspnoea scale as a measure of disability in patients with chronic obstructive pulmonary disease. *Thorax.* 1999;54(7):581-586.

**20.** Binder EF, Miller JP, Ball LJ. Development of a test of physical performance for the nursing home setting. *Gerontologist.* 2001;41(5):671-679.

**21.** Bouchard C, Tremblay A, Leblanc C, Lortie G, Savard R, Theriault G. A method to assess energy expenditure in children and adults. *The American Journal of Clinical Nutrition.* 1983;37(3):461-467.

**22.** Boult C, Krinke UB, Urdangarin CF, Skarin V. The validity of nutritional status as a marker for future disability and depressive symptoms among high-risk older adults. *Journal of the American Geriatrics Society.* 1999;47(8):995-999.

**23.** Bowns I, Challis D, Tong MS. Case finding in elderly people: validation of a postal questionnaire. *British Journal of General Practice.* 1991;41(344):100-104.

**24.** Braido F, Baiardini I, Tarantini F, et al. Chronic cough and QoL in allergic and respiratory diseases measured by a new specific validated tool-CCIQ. *Journal of Investigational Allergology & Clinical Immunology.* 2006;16(2):110-116.

**25.** Budzynski HK, Budzynski T. Perceived Physical Functioning Scale for community dwelling elderly... 34th Annual Communicating Nursing Research Conference/15th Annual WIN Assembly, "Health Care Challenges Beyond 2001: Mapping the Journey for Research and Practice," held April 19-21, 2001 in Seattle, Washington. *Communicating nursing research.* 2001;34(Journal Article):325-325.

**26.** Burckhardt CS, Woods SL, Schultz AA, Ziebarth DM. Quality of life of adults with chronic illness: a psychometric study. *Research in nursing & health.* 1989;12(6):347-354.

**27.** Carter R, Holiday DB, Grothues C, Nwasuruba C, Stocks J, Tiep B. Criterion validity of the Duke Activity Status Index for assessing functional capacity in patients with chronic obstructive pulmonary disease. *Journal of cardiopulmonary rehabilitation.* 2002;22(4):298-308.

**28.** Cartmel B, Moon TE. Comparison of two physical activity questionnaires, with a diary, for assessing physical activity in an elderly population. *Journal of clinical epidemiology.* 1992;45(8):877-883.

**29.** Chasan-Taber S, Rimm EB, Stampfer MJ, et al. Reproducibility and Validity of a Self-Administered Physical Activity Questionnaire for Male Health Professionals. *Epidemiology.* 1996;7(1):81-86.

**30.** Chen H, Eisner MD, Katz PP, Yelin EH, Blanc PD. Measuring disease-specific quality of life in obstructive airway disease: Validation of a modified version of the airways questionnaire 20. *Chest.* 2006;129(6):1644.

**31.** Chen Q, Kane RL. Effects of using consumer and expert ratings of an activities of daily living scale on predicting functional outcomes of postacute care. *Journal of clinical epidemiology.* 2001;54(4):334-342.

**32.** Chester GA. *Normative data for the brief symptom inventory for mature and independent living adults*2001.

**33.** Chiou C. Development and psychometric assessment of the Physical Symptom Distress Scale. *Journal of Pain & Symptom Management.* 1998;16(2):87-95.

**34.** Choi YH, Kim MS, Byon YS, Won JS. [Health status of elderly persons in Korea]. *Kanho Hakhoe Chi [Journal of Nurses Academic Society].* 1990;20(3):307-323.

**35.** Clarke JE, Eccleston C. Assessing the quality of walking in adults with chronic pain: the development and preliminary psychometric evaluation of the Bath Assessment of Walking Inventory. *European Journal of Pain: Ejp.* 2009;13(3):305-311.

**36.** Coleman EA, Wagner EH, Grothaus LC, Hecht J, Savarino J, Buchner DM. Predicting hospitalization and functional decline in older health plan enrollees: are administrative data as accurate as self-report?[see comment]. *Journal of the American Geriatrics Society.* 1998;46(4):419-425.

**37.** Colombel JF, Yazdanpanah Y, Laurent F, Houcke P, Delas N, Marquis P. [Quality of life in chronic inflammatory bowel diseases. Validation of a questionnaire and first French data]. *Gastroenterologie clinique et biologique.* 1996;20(12):1071-1077.

**38.** Covinsky KE, Hilton J, Lindquist K, Dudley RA. Development and validation of an index to predict activity of daily living dependence in community-dwelling elders. *Medical care.* 2006;44(2):149-157.

**39.** Covinsky KE, Palmer RM, Counsell SR, Pine ZM, Walter LC, Chren M. Functional status before hospitalization in acutely ill older adults: Validity and clinical importance of retrospective reports. *Journal of the American Geriatrics Society.* 2000;48(2):164-169.

**40.** Craig CL, Marshall AL, Sjostrom M, et al. International physical activity questionnaire: 12-country reliability and validity. *Medicine and science in sports and exercise.* 2003;35(8):1381-1395.

**41.** Crawford B, Monz B, Hohlfeld J, et al. Development and validation of a cough and sputum assessment questionnaire. *Respiratory medicine.* 2008;102(11):1545-1555.

**42.** Creel GL, Light KE, Thigpen MT. Concurrent and construct validity of scores on the Timed Movement Battery. *Physical Therapy.* 2001;81(2):789-798.

**43.** Crockett DJ, Tuokko H, Koch W, Parks R. The assessment of everyday functioning using the Present Functioning Questionnaire and the Functional Rating Scale in elderly samples. *Clinical gerontologist.* 1989;8(3):3-25.

**44.** Crouch MJ. *Assessing components of loss-related dysfunction in the elderly*2003.

**45.** Cullum CM, Saine K, Chan LD, Martin-Cook K, Gray KF, Weiner MF. Performance-Based instrument to assess functional capacity in dementia: The Texas Functional Living Scale. *Neuropsychiatry, Neuropsychology, & Behavioral Neurology.* 2001;14(2):103-108.

**46.** Dahlin-Ivanoff S, Sonn U, Svensson E. Development of an ADL instrument targeting elderly persons with age-related macular degeneration. *Disability & Rehabilitation.* 2001;23(2):69-79.

**47.** Daltroy LH, Phillips CB, Eaton HM, et al. Objectively measuring physical ability in elderly persons: the Physical Capacity Evaluation. *American Journal of Public Health.* 1995;85(4):558-560.

**48.** De Leo D, Diekstra RF, Lonnqvist J, et al. LEIPAD, an internationally applicable instrument to assess quality of life in the elderly. *Behavioral Medicine.* 1998;24(1):17-27.

**49.** de Veer AJ, de Bakker DH. Measuring unmet needs to assess the quality of home health care. *International Journal for Quality in Health Care.* 1994;6(3):267-274.

**50.** Deniston OL, Jette A. A functional status assessment instrument: validation in an elderly population. *Health services research.* 1980;15(1):21-34.

**51.** Devins GM. Using the illness intrusiveness ratings scale to understand health-related quality of life in chronic disease. *Journal of psychosomatic research.* 2009(Journal Article).

**52.** Devins GM, Binik YM, Hutchinson TA, Hollomby DJ, Barre PE, Guttmann RD. The emotional impact of end-stage renal disease: importance of patients' perception of intrusiveness and control. *International journal of psychiatry in medicine.* 1983;13(4):327-343.

**53.** Devlen J MSMP. Measuring Quality of Life: A Disease-Specific Approach.

**54.** Deyo RA, Inui TS, Leininger JD, Overman SS. Measuring functional outcomes in chronic disease: a comparison of traditional scales and a self-administered health status questionnaire in patients with rheumatoid arthritis. *Medical care.* 1983;21(2):180-192.

**55.** Dickerson AE, Fisher AG. Culture-relevant functional performance assessment of the Hispanic elderly. *Occupational Therapy Journal of Research.* 1995;15(1):50-68.

**56.** Dinger MK, Oman RF, Taylor EL, Vesely SK, Able J. Stability and convergent validity of the Physical Activity Scale for the Elderly (PASE). *Journal of Sports Medicine & Physical Fitness.* 2004;44(2):186-192.

**57.** Dixon D, Pollard B, Johnston M. What does the chronic pain grade questionnaire measure? *Pain.* 2007;130(3):249-253.

**58.** Doble SE, Fisher AG. The dimensionality and validity of the Older Americans Resources and Services (OARS) Activities of Daily Living (ADL) Scale. *Journal of outcome measurement.* 1998;2(1):4-24.

**59.** DuBose KD, Edwards S, Ainsworth BE, Reis JP, Slattery ML. Validation of a historical physical activity questionnaire in middle-aged women. *Journal of Physical Activity & Health.* 2007;4(3):343-355.

**60.** Duiverman ML, Wempe JB, Bladder G, Kerstjens HA, Wijkstra PJ. Health-related quality of life in COPD patients with chronic respiratory failure. *The European respiratory journal : official journal of the European Society for Clinical Respiratory Physiology.* 2008;32(2):379.

**61.** Eakman AM. *A reliability and validity study of the Meaningful Activity Participation Assessment*2008.

**62.** Eaton T, Young P, Fergusson W, Garrett JE, Kolbe J. The Dartmouth COOP Charts: A simple, reliable, valid and responsive quality of life tool for chronic obstructive pulmonary disease. *Quality of Life Research: An International Journal of Quality of Life Aspects of Treatment, Care & Rehabilitation.* 2005;14(3):577-585.

**63.** Edwards R, Telfair J, Cecil H, Lenoci J. Reliability and validity of a self-efficacy instrument specific to sickle cell disease. *Behaviour Research & Therapy.* 2000;38(9):951-963.

**64.** Eisner MD, Trupin L, Katz PP, et al. Development and validation of a survey-based COPD severity score. *Chest.* 2005;127(6):1890.

**65.** Fabris F MMPMFGVPSC. Dependance medical index (DMI) in elderly persons: a tool for identification of dependence for medical reasons. *Bold.* 1996;6(Journal Article):9-12.

**66.** Fairbank JC, Couper J, Davies JB, O'Brien JP. The Oswestry low back pain disability questionnaire. *Physiotherapy.* 1980;66(8):271-273.

**67.** Feitel B. A checklist for measuring nonfunctional behavior of regressed chronic psychiatric patients. *Journal of clinical psychology.* 1981;37(1):158-160.

**68.** Fillenbaum GG, Chandra V, Ganguli M, et al. Development of an activities of daily living scale to screen for dementia in an illiterate rural older population in India. *Age & Ageing.* 1999;28(2):161-168.

**69.** Fillenbaum GG, Pfeiffer E. The Mini-Mult: A cautionary note. *Journal of consulting and clinical psychology.* 1976;44(5):698-703.

**70.** Fine MA, Tangeman PJ. Adaptive Behavior Scale predictive validity with elderly male veterans. *Clinical gerontologist.* 1993;14(2):27-31.

**71.** Fisher AG. The assessment of IADL motor skills: an application of many-faceted Rasch analysis. *The American journal of occupational therapy.: official publication of the American Occupational Therapy Association.* 1993;47(4):319-329.

**72.** Floyd FJ, Haynes SN, Doll ER, et al. Assessing retirement satisfaction and perceptions of retirement experiences. *Psychology and aging.* 1992;7(4):609-621.

**73.** Frederiks CMA, te Wierik MJM, Visser AP, Sturmans F. A scale for the functional status of the elderly living at home. *Journal of advanced nursing.* 1991;16(3):287-292.

**74.** Gabel CP, Michener LA, Burkett B, Neller A. The Upper Limb Functional Index: development and determination of reliability, validity, and responsiveness. *Journal of hand therapy : official journal of the American Society of Hand Therapists.* 2006;19(3):328-348; quiz 349.

**75.** Gerety MB, Mulrow CD, Tuley MR, et al. Development and validation of a physical performance instrument for the functionally impaired elderly: the Physical Disability Index (PDI). *Journal of gerontology.* 1993;48(2):M33-38.

**76.** Gosman-Hedstrom G, Svensson E. Parallel reliability of the functional independence measure and the Barthel ADL index. *Disability & Rehabilitation.* 2000;22(16):702-715.

**77.** Granger Carl V MD, Benjamin D. Wright P. Looking ahead to the use of functional assessment in ambulatory physiatric and primary care*.* Vol 41993:595-605.

**78.** Granger CV, Ottenbacher KJ, Baker JG, Sehgal A. Reliability of a brief outpatient functional outcome assessment measure. *American Journal of Physical Medicine & Rehabilitation / Association of Academic Physiatrists.* 1995;74(6):469-475.

**79.** Greenland P, Ries AL, Williams MA. Literature update: selected abstracts from recent publications in cardiac and pulmonary disease prevention, rehabilitation, and exercise physiology. [Commentary on] Development and validation of a standardized measure of activity of daily living in patients with severe chronic obstructive pulmonary disease: the London Chest Activity of Daily Living Scale. *Journal of cardiopulmonary rehabilitation.* 2001;21(3):178-179.

**80.** Gulick EE. Reliability and validity of the work assessment scale for persons with multiple sclerosis. *Nursing research.* 1991;40(2):107-112.

**81.** Gulick EE, Yam M, Touw MM. Work performance by persons with multiple sclerosis: conditions that impede or enable the performance of work. *International journal of nursing studies.* 1989;26(4):301-311.

**82.** Haapaniemi TH, Sotaniemi KA, Sintonen H, Taimela E. The generic 15D instrument is valid and feasible for measuring health related quality of life in Parkinson's disease. *Journal of Neurology, Neurosurgery & Psychiatry.* 2004;75(7):976-983.

**83.** Hagg O, Fritzell P, Romberg K, Nordwall A. The General Function Score: a useful tool for measurement of physical disability. Validity and reliability. *European spine journal : official publication of the European Spine Society, the European Spinal Deformity Society, and the European Section of the Cervical Spine Research Society.* 2001;10(3):203-210.

**84.** Han CW, Yajima Y, Lee EJ, et al. Development and construct validation of the Korean competence scale (KCS). *Tohoku Journal of Experimental Medicine.* 2004;203(4):331-337.

**85.** Harada K, Ota A, Shibata A, Oka K, Nakamura Y, Muraoka I. Development of the exercise-specified subjective health status scale for the frail elderly... 7th World Congress on Aging and Physical Activity. *Journal of Aging & Physical Activity.* 2008;16(Journal Article):S185-S185.

**86.** Harwood RH, Rogers A, Dickinson E, Ebrahim S. Measuring handicap: the London Handicap Scale, a new outcome measure for chronic disease. *Quality in Health Care.* 1994;3(1):11-16.

**87.** Hebert R, Carrier R, Bilodeau A. The Functional Autonomy Measurement System (SMAF): description and validation of an instrument for the measurement of handicaps. *Age & Ageing.* 1988;17(5):293-302.

**88.** Hidalgo JL, Gras CB, Lapeira JM, et al. The Hearing-Dependent Daily Activities Scale to evaluate impact of hearing loss in older people. *Annals of Family Medicine.* 2008;6(5):441-447.

**89.** Hiratsuka T, Kida K. Quality of life measurements using a linear analog scale for elderly patients with chronic lung disease. *Internal medicine (Tokyo, Japan).* 1993;32(11):832-836.

**90.** Hodgev V, Kostianev S, Marinov B. University of Cincinnati Dyspnea Questionnaire for Evaluation of Dyspnoea during physical and speech activities in patients with chronic obstructive pulmonary disease: a validation analysis. *Clinical Physiology & Functional Imaging.* 2003;23(5):269-274.

**91.** Holm I, Friis A, Storheim K, Brox JI. Measuring self-reported functional status and pain in patients with chronic low back pain by postal questionnaires: a reliability study. *Spine.* 2003;28(8):828-833.

**92.** Huijbregts MP, Teare GF, McCullough C, et al. Standardization of the continuing care activity measure: a multicenter study to assess reliability, validity, and ability to measure change. *Physical Therapy.* 2009;89(6):546-555.

**93.** Iida N, Kohashi N, Koyama W. [The reliability and validity of a new self-completed questionnaire (QUIK)]. *Nippon Ronen Igakkai Zasshi - Japanese Journal of Geriatrics.* 1995;32(2):96-100.

**94.** Incalzi RA, Corsonello A, Pedone C, et al. Construct validity of activities of daily living scale: a clue to distinguish the disabling effects of COPD and congestive heart failure. *Chest.* 2005;127(3):830-838.

**95.** Itzkovich M, Catz A, Tamir A, et al. Spinal pain independence measure--a new scale for assessment of primary ADL dysfunction related to LBP. *Disability & Rehabilitation.* 2001;23(5):186-191.

**96.** Jensen MP, Turner JA, Romano JM, Strom SE. The Chronic Pain Coping Inventory: development and preliminary validation. *Pain.* 1995;60(2):203-216.

**97.** Jette AM, Davies AR, Cleary PD, et al. The Functional Status Questionnaire: reliability and validity when used in primary care. *Journal of general internal medicine.* 1986;1(3):143-149.

**98.** Kai I, Ohi G, Kobayashi Y, Ishizaki T, Hisata M, Kiuchi M. Quality of life: a possible health index for the elderly. *Asia-Pacific Journal of Public Health.* 1991;5(3):221-227.

**99.** Kames LD, Naliboff BD, Heinrich RL, Schag CC. The chronic illness problem inventory: problem-oriented psychosocial assessment of patients with chronic illness. *International journal of psychiatry in medicine.* 1984;14(1):65-75.

**100.** Katsura H, Yamada K, Kida K. Usefulness of a linear analog scale questionnaire to measure health-related quality of life in elderly patients with chronic obstructive pulmonary disease. *Journal of the American Geriatrics Society.* 2003;51(8):1131-1135.

**101.** Katz JN, Wright EA, Baron JA, Losina E. Development and validation of an index of musculoskeletal functional limitations. *BMC Musculoskeletal Disorders.* 2009;10(Journal Article):62.

**102.** Katz S, Ford AB, Moskowitz RW, Jackson BA, Jaffe MW. Studies of Illness in the Aged. the Index of Adl: a Standardized Measure of Biological and Psychosocial Function. *JAMA : the journal of the American Medical Association.* 1963;185(Journal Article):914-919.

**103.** Kincannon JC. Prediction of the standard MMPI scale scores from 71 items: the mini-mult. *Journal of consulting and clinical psychology.* 1968;32(3):319-325.

**104.** Lachman ME, Howland J, Tennstedt S, Jette A, Assmann S, Peterson EW. Fear of falling and activity restriction: the survey of activities and fear of falling in the elderly (SAFE). *Journals of Gerontology Series B-Psychological Sciences & Social Sciences.* 1998;53(1):P43-50.

**105.** Larson JL, Kapella MC, Wirtz S, Covey MK, Berry J. Reliability and validity of the Functional Performance Inventory in patients with moderate to severe chronic obstructive pulmonary disease. *Journal of nursing measurement.* 1998;6(1):55-73.

**106.** Lawton MP, Brody EM. Assessment of older people: self-maintaining and instrumental activities of daily living. *The Gerontologist.* 1969;9(3):179-186.

**107.** Leeuw M, Goossens ME, van Breukelen GJ, Boersma K, Vlaeyen JW. Measuring perceived harmfulness of physical activities in patients with chronic low back pain: the Photograph Series of Daily Activities--short electronic version. *Journal of Pain.* 2007;8(11):840-849.

**108.** Leidy NK. Using functional status to assess treatment outcomes. *Chest.* 1994;106(6):1645-1646.

**109.** Leidy NK. Functional status and the forward progress of merry-go-rounds: toward a coherent analytical framework. *Nursing research.* 1994;43(4):196-202.

**110.** Leidy NK, Knebel AR. Clinical validation of the functional performance inventory in patients with chronic obstructive pulmonary disease. *Respiratory care.* 1999;44(8):932.

**111.** Leidy NK, Schmier JK, Jones MK, Lloyd J, Rocchiccioli K. Evaluating symptoms in chronic obstructive pulmonary disease: validation of the Breathlessness, Cough and Sputum Scale. *Respiratory medicine.* 2003;97(Suppl A):S59-70.

**112.** Lennon S, Johnson L. The modified rivermead mobility index: validity and reliability. *Disability & Rehabilitation.* 2000;22(18):833-839.

**113.** Lenze EJ, Munin MC, Quear T, et al. The Pittsburgh Rehabilitation Participation Scale: reliability and validity of a clinician-rated measure of participation in acute rehabilitation. *Archives of Physical Medicine & Rehabilitation.* 2004;85(3):380-384.

**114.** Letts L, Scott S, Burtney J, Marshall L, McKean M. The reliability and validity of the safety assessment of function and the environment for rehabilitation (SAFER tool). *British Journal of Occupational Therapy.* 1998;61(3):127-132.

**115.** Leung AS, Chan KK, Sykes K, Chan KS. Reliability, validity, and responsiveness of a 2-min walk test to assess exercise capacity of COPD patients. *Chest.* 2006;130(1):119-125.

**116.** Levine S, Gillen M, Weiser P, Feiss G, Goldman M, Henson D. Inspiratory pressure generation: comparison of subjects with COPD and age-matched normals. *Journal of applied physiology.* 1988;65(2):888-899.

**117.** Lincoln NB, Gladman JR. The Extended Activities of Daily Living scale: a further validation. *Disability and rehabilitation.* 1992;14(1):41-43.

**118.** Linn MW, Linn BS. Self-evaluation of life function (self) scale: a short, comprehensive self-report of health for elderly adults. *Journal of gerontology.* 1984;39(5):603-612.

**119.** Linzer M, Gold DT, Pontinen M, Divine GW, Felder A, Brooks WB. Recurrent syncope as a chronic disease: preliminary validation of a disease-specific measure of functional impairment. *Journal of General Internal Medicine.* 1994;9(4):181-186.

**120.** Littman AJ, White E, Kristal AR, Patterson RE, Satia-Abouta J, Potter JD. Assessment of a one-page questionnaire on long-term recreational physical activity. *Epidemiology.* 2004;15(1):105-113.

**121.** Livingston G, Watkin V, Manela M, Rosser R, Katona C. Quality of life in older people. *Aging & Mental Health.* 1998;2(1):20-23.

**122.** Ljungquist T, Nygren A, Jensen I, Harms-Ringdahl K. Physical performance tests for people with spinal pain--sensitivity to change. *Disability & Rehabilitation.* 2003;25(15):856-866.

**123.** Lundin-Olsson L, Nyberg L, Gustafson Y. Attention, frailty, and falls: the effect of a manual task on basic mobility. *Journal of the American Geriatrics Society.* 1998;46(6):758-761.

**124.** Macfarlane DJ, Chou KL, Cheng YH, Chi I. Validity and normative data for thirty-second chair stand test in elderly community-dwelling Hong Kong Chinese. *American Journal of Human Biology.* 2006;18(3):418-421.

**125.** MacKenzie CR, Charlson ME, DiGioia D, Kelley K. A patient-specific measure of change in maximal function. *Archives of Internal Medicine.* 1986;146(7):1325-1329.

**126.** MacKnight C, Rockwood K. A Hierarchical Assessment of Balance and Mobility. *Age & Ageing.* 1995;24(2):126-130.

**127.** Maeda A, Yuasa T, Nakamura K, Higuchi S, Motohashi Y. Physical performance tests after stroke: reliability and validity. *American Journal of Physical Medicine & Rehabilitation.* 2000;79(6):519-525.

**128.** Magnussen L, Strand LI, Lygren H. Reliability and validity of the back performance scale: observing activity limitation in patients with back pain. *Spine.* 2004;29(8):903-907.

**129.** Mahoney RI, Barthel DW. Surya Shah, PhD, OTD, MEd, OTR, FAOTA, Professor Occupational Therapy and Neurology, Visiting Professor Neurorehabilitation, University of Tennessee Health Sciences Center, 930 Madison, Suite 601, Memphis, TN 38163; 1965:1 p-1 p.

**130.** Mahurin RK, DeBettignies BH, Pirozzolo FJ. Structured assessment of independent living skills: preliminary report of a performance measure of functional abilities in dementia. *Journal of gerontology.* 1991;46(2):P58-66.

**131.** Majani G, Callegari S, Pierobon A, Giardini A, Vidotto G. Satisfaction profile (SAT-P): A new evaluation instrument in a clinical environment. *Psicoterapia Cognitiva e Comportamentale.* 1997;3(1):27-41.

**132.** Majani G CAPA. A New Instrument in Quality-of-Lie Assessment The Satisfaction Profile (SAT-P). *International Journal of Mental Health.* 1999;28(3):77-82.

**133.** Marcus BH, Rossi JS, Selby VC, Niaura RS, Abrams DB. The stages and processes of exercise adoption and maintenance in a worksite sample. *Health psychology : official journal of the Division of Health Psychology, American Psychological Association.* 1992;11(6):386-395.

**134.** Marks GB, Dunn SM, Woolcock AJ. A scale for the measurement of quality of life in adults with asthma. *Journal of clinical epidemiology.* 1992;45(5):461-472.

**135.** Marquis P. Evaluation de l'impact de l'artériopathie oblitérante des membres inférieurs sur la qualité de vie. *Drugs.* 1998;56(6):25-35.

**136.** Marquis P, Fayol C, McCarthy C, Fiessinger JN. Measurement of quality of life in intermittent claudication. Clinical validation of a questionnaire. *Presse medicale (Paris, France : 1983).* 1994;23(28):1288-1292.

**137.** Martin LL. Validity and reliability of a quality-of-life instrument: the chronic respiratory disease questionnaire. *Clinical nursing research.* 1994;3(2):146-156.

**138.** Martinez FJ, Raczek AE, Seifer FD, et al. Development and Initial Validation of a Self-Scored COPD Population Screener Questionnaire (COPD-PS). *COPD: Journal of Chronic Obstructive Pulmonary Disease.* 2008;5(2):85-95.

**139.** McCabe MA, Granger CV. Content validity of a pediatric functional independence measure. *Applied Nursing Research : ANR.* 1990;3(3):120-122.

**140.** McGee MA, Johnson AL, Kay DW. The description of activities of daily living in five centres in England and Wales. Medical Research Council Cognitive Function and Ageing Study. *Age & Ageing.* 1998;27(5):605-613.

**141.** Medinas-Amoros M, Alorda C, Renom F, et al. Quality of life in patients with chronic obstructive pulmonary disease: the predictive validity of the BODE index. *Chronic Respiratory Disease.* 2008;5(1):7-11.

**142.** Mendelsohn ME, Connelly DM, Overend TJ, Petrella RJ. Validity of values for metabolic equivalents of task during submaximal all-extremity exercise and reliability of exercise responses in frail older adults. *Physical Therapy.* 2008;88(6):747-756.

**143.** Mezzani A, Corra U, Baroffio C, Bosimini E, Giannuzzi P. Habitual activities and peak aerobic capacity in patients with asymptomatic and symptomatic left ventricular dysfunction. *Chest.* 2000;117(5):1291-1299.

**144.** Mielenz T, Jackson E, Currey S, DeVellis R, Callahan LF. Psychometric properties of the Centers for Disease Control and Prevention Health-Related Quality of Life (CDC HRQOL) items in adults with arthritis. *Health & Quality of Life Outcomes.* 2006;4(Journal Article):66.

**145.** Mihay L, Iltzsche E, Tribby A, et al. Balance and perceived confidence with performance of instrumental activities of daily living: a pilot study of Tai Chi inspired exercise with elderly retirement-community dwellers. *Physical & Occupational Therapy in Geriatrics.* 2003;21(3):75-86.

**146.** Mithal M, Mann WC, Granger CV. The role of coronary heart disease (CHD) in functional limitation in community dwelling elders. *Physical & Occupational Therapy in Geriatrics.* 2001;19(3):33-46.

**147.** Mjm, de Jong N, Schouten EG, van Staveren WA, Kok FJ. Physical exercise or micronutrient supplementation for the wellbeing of the frail elderly? A randomised controlled trial. *British journal of sports medicine.* 2002;36(2):126-131.

**148.** Mobily KE, Lemke JH, Ostiguy LJ, Woodard RJ. Leisure repertoire in a sample of midwestern elderly: The case for exercise. *Journal of Leisure Research.* 1993;25(1):84-99.

**149.** Moore DS, Ellis R, Allen PD, et al. Construct validation of physical activity surveys in culturally diverse older adults: a comparison of four commonly used questionnaires. *Research Quarterly for Exercise & Sport.* 2008;79(1):42-50.

**150.** Moore R, Berlowitz D, Denehy L, Jackson B, McDonald CF. Comparison of pedometer and activity diary for measurement of physical activity in chronic obstructive pulmonary disease. *Journal of Cardiopulmonary Rehabilitation and Prevention.* 2009;29(1):57.

**151.** Morgan K, Clarke D. Customary physical activity and survival in later life: a study in Nottingham, UK. *Journal of Epidemiology & Community Health.* 1997;51(5):490-493.

**152.** Morio B, Barra V, Ritz P, et al. Benefit of endurance training in elderly people over a short period is reversible. *European journal of applied physiology.* 2000;81(4):329-336.

**153.** Motohashi Y, Maeda A, Yuasa T, Higuchi S. Reliability and validity of the questionnaire to determine the biosocial rhythms of daily living in the disabled elderly. *Journal of Physiological Anthropology & Applied Human Science.* 2000;19(6):263-269.

**154.** Muhlig S, Bergmann KC, Emmermann E, Petermann F. "Questionnaire on Quality of Life in Asthma"--studies of the dimensionality and references for evaluation. *Pneumologie (Stuttgart, Germany).* 1998;52(1):35-40.

**155.** Muhlig S, Petermann F. Hints on assessment instruments for quality assurance in rehabilitation - No. 15. Disease specific instruments for assessing quality of life in patients with asthma and chronic obstructive pulmonary disease. *Hinweise auf bewertungsinstrumente zur qualitatssicherung in der rehabilitation - Blatt 15. Krankheitsspezifische erhebungsverfahren zur lebensqualitat bei patienten mit asthma und chronisch-obstruktiver bronchitis.* 1998;37(3):XXV; XXXVIII-XXV; XXXVIII.

**156.** Murray M, Lefort S, Ribeiro V. The SF-36: reliable and valid for the institutionalized elderly? *Aging & Mental Health.* 1998;2(1):24-27.

**157.** Nagamatsu T, Oida Y, Kitabatake Y, et al. A 6-year cohort study on relationship between functional fitness and impairment of ADL in community-dwelling older persons. *Journal of Epidemiology.* 2003;13(3):142-148.

**158.** Nakagaichi M, Tanaka K. Development of a 12-min treadmill walk test at a self-selected pace for the evaluation of cardiorespiratory fitness in adult men. *Applied Human Science.* 1998;17(6):281-288.

**159.** Nakazato K, Shimonaka Y, Narita K, Honjo Y. [Development of behavior rating scale for the elderly]. *Nippon Ronen Igakkai Zasshi - Japanese Journal of Geriatrics.* 1991;28(6):790-800.

**160.** Nguyen HQ, Steele B, Benditt JO. Use of accelerometers to characterize physical activity patterns with COPD exacerbations. *International Journal of Copd.* 2006;1(4):455-460.

**161.** Nielson WR, Jensen MP, Hill ML. An activity pacing scale for the chronic pain coping inventory: development in a sample of patients with fibromyalgia syndrome. *Pain.* 2001;89(2-3):111-115.

**162.** Nieves JW, Zion M, Pahor M, et al. Evaluation of continuous summary physical performance scores (CSPPS) in an elderly cohort. *Aging-Clinical & Experimental Research.* 2005;17(3):193-200.

**163.** Nikolaus T, Specht-Leible N, Bach M, Oster P, Schlierf G. [Social aspects in diagnosis and therapy of very elderly patients. Initial experiences with a newly developed questionnaire within the scope of geriatric assessment]. *Zeitschrift fur Gerontologie.* 1994;27(4):240-245.

**164.** Ninot G, Moullec G, Desplan J, Prefaut C, Varray A. Daily functioning of dyspnea, self-esteem and physical self in patients with moderate COPD before, during and after a first inpatient rehabilitation program. *Disability & Rehabilitation.* 2007;29(22):1671-1678.

**165.** Oakley F, Sunderland T, Hill JL, Phillips SL. The Daily Activities Questionnaire: A functional assessment for people with Alzheimer's disease. *Physical & Occupational Therapy in Geriatrics.* 1991;10(2):67-81.

**166.** Ohta T, Haga H, Osada H, et al. [Development and evaluation of a QOL questionnaire for elderly subject living in a community]. *Nippon Koshu Eisei Zasshi - Japanese Journal of Public Health.* 2001;48(4):258-267.

**167.** Oida Y, Kitabatake Y, Nishijima Y, et al. Effects of a 5-year exercise-centered health-promoting programme on mortality and ADL impairment in the elderly. *Age & Ageing.* 2003;32(6):585-592.

**168.** Okochi J, Toba K, Takahashi T, et al. Simple screening test for risk of falls in the elderly. *Geriatrics & Gerontology International.* 2006;6(4):223-227.

**169.** Okochi J, Utsunomiya S, Takahashi T. Health measurement using the ICF: test-retest reliability study of ICF codes and qualifiers in geriatric care. *Health & Quality of Life Outcomes.* 2005;3(Journal Article):46.

**170.** Oliver R, Blathwayt J, Brackley C, Tamaki T. Development of the Safety Assessment of Function and the Environment for Rehabilitation (SAFER) tool. *Canadian journal of occupational therapy.Revue canadienne d'ergotherapie.* 1993;60(2):78-82.

**171.** Oliveri S, Carpenter IG, Demopoulos G. Validity and reliability of the Winchester Disability Rating Scale (2): A comprehensive screening instrument for the elderly in the community. *Gerontology.* 1994;40(6):319-324.

**172.** Orfila F, Ferrer M, Lamarca R, Tebe C, Domingo-Salvany A, Alonso J. Gender differences in health-related quality of life among the elderly: the role of objective functional capacity and chronic conditions. *Social science & medicine.* 2006;63(9):2367-2380.

**173.** Orsini N, Bellocco R, Bottai M, et al. Profile of physical activity behaviors among Swedish women aged 56-75 years. *Scandinavian journal of medicine & science in sports.* 2008;18(1):95-101.

**174.** Oschutz H, Weisser B, Finck M, Hurtmanns J, Schaller HJ. Development of a walking stage test (PWT) for the elderly. *Zeitschrift fur Gerontologie und Geriatrie.* 2005;38(2):139-150.

**175.** Paier GS. *Development and testing of an instrument to assess functional status in the elderly*, UNIVERSITY OF PENNSYLVANIA; 1994.

**176.** Pan AM, Stiell IG, Clement CM, Acheson J, Aaron SD. Feasibility of a structured 3-minute walk test as a clinical decision tool for patients presenting to the emergency department with acute dyspnoea. *Emergency Medicine Journal.* 2009;26(4):278-282.

**177.** Panagiotakos DB, Polystipioti A, Polychronopoulos E. Prevalence of type 2 diabetes and physical activity status in elderly men and women from Cyprus (The MEDIS Study). *Asia-Pacific Journal of Public Health.* 2007;19(3):22-28.

**178.** Patel SA, Benzo RP, Slivka WA, Sciurba FC. Activity monitoring and energy expenditure in COPD patients: a validation study. *Copd: Journal of Chronic Obstructive Pulmonary Disease.* 2007;4(2):107-112.

**179.** Patel SA, Sciurba FC. Emerging concepts in outcome assessment for COPD clinical trials. *Seminars in Respiratory & Critical Care Medicine.* 2005;26(2):253-262.

**180.** Patrick JM, Bassey EJ, Irving JM, Blecher A, Fentem PH. Objective measurements of customary physical activity in elderly men and women before and after retirement. *Quarterly Journal of Experimental Physiology.* 1986;71(1):47-58.

**181.** Patterson MB, Mack JL. The Cleveland Scale for Activities of Daily Living (CSADL): Its reliability and validity. *Journal of Clinical Geropsychology.* 2001;7(1):15-28.

**182.** Peloquin L, Gauthier P, Bravo G, Lacombe G, Billiard J. Reliability and validity of the five-minute walking field test for estimating VO2 peak in elderly subjects with knee osteoarthritis. *Journal of Aging & Physical Activity.* 1998;6(1):36-44.

**183.** Pfeiffer E, Johnson TM, Chiofolo RC. Functional assessment of elderly subjects in four service settings. *Journal of the American Geriatrics Society.* 1981;29(10):433-437.

**184.** Pieper B, Templin TN, Birk TJ, Kirsner RS. Chronic venous disorders and injection drug use: impact on balance, gait, and walk speed. *Journal of Wound, Ostomy, & Continence Nursing.* 2008;35(3):301-310.

**185.** Pine ZM, Gurland B, Chren MM. Report of having slowed down: evidence for the validity of a new way to inquire about mild disability in elders. *Journals of Gerontology Series A-Biological Sciences & Medical Sciences.* 2000;55(7):M378-383.

**186.** Pinto-Plata VM, Celli-Cruz RA, Vassaux C, et al. Differences in cardiopulmonary exercise test results by American Thoracic Society/European Respiratory Society-Global Initiative for Chronic Obstructive Lung Disease stage categories and gender. *Chest.* 2007;132(4):1204-1211.

**187.** Podsiadlo D, Richardson S. The timed "Up & Go": a test of basic functional mobility for frail elderly persons. *Journal of the American Geriatrics Society.* 1991;39(2):142-148.

**188.** Pollak N, Rheault W, Stoecker JL. Reliability and validity of the FIM for persons aged 80 years and above from a multilevel continuing care retirement community. *Archives of Physical Medicine & Rehabilitation.* 1996;77(10):1056-1061.

**189.** Pols MA, Peeters PH, Kemper HC, Collette HJ. Repeatability and relative validity of two physical activity questionnaires in elderly women. *Medicine & Science in Sports & Exercise.* 1996;28(8):1020-1025.

**190.** Pols MA, Peeters PH, Ocke MC, et al. Relative validity and repeatability of a new questionnaire on physical activity. *Preventive medicine.* 1997;26(1):37-43.

**191.** Pomeroy V. Development of an ADL oriented assessment-of-mobility scale suitable for use with elderly people with dementia. *Physiotherapy.* 1990;76(8):446-448.

**192.** Powell LE, Myers AM. The Activities-specific Balance Confidence (ABC) Scale. *The journals of gerontology.Series A, Biological sciences and medical sciences.* 1995;50A(1):M28-34.

**193.** Pransky G, Feuerstein M, Himmelstein J, Katz JN, Vickers-Lahti M. Measuring functional outcomes in work-related upper extremity disorders. Development and validation of the Upper Extremity Function Scale. *Journal of Occupational & Environmental Medicine.* 1997;39(12):1195-1202.

**194.** Provenier F, Jordaens L. Evaluation of six minute walking test in patients with single chamber rate responsive pacemakers. *British heart journal.* 1994;72(2):192-196.

**195.** Puhan M. [Severity of COPD--a broader prognostic assessment is necessary]. *Praxis.* 2007;96(48):1901-1905.

**196.** Quirk FH, Jones PW. Patients' perception of distress due to symptoms and effects of asthma on daily living and an investigation of possible influential factors. *Clinical science (London, England : 1979).* 1990;79(1):17-21.

**197.** Reardon JZ, Lareau SC, ZuWallack R. Functional status and quality of life in chronic obstructive pulmonary disease.[see comment]. *American Journal of Medicine.* 2006;119(10 Suppl 1):32-37.

**198.** Recchia G, de Carli G, Parise G, Zerbini O. Quality of life: A new approach to the assessment of the pharmacological treatment in the elderly patient. *Archives of Gerontology and Geriatrics.* 1991;Suppl 2(Journal Article):33-37.

**199.** Reid D.W ZM. A survey of the reinforcements and activities elderly citizens feel are important for their general happiness. *Essence.* 1977;2(Journal Article):5-24.

**200.** Rejeski WJ, Brawley LR, Ambrosius WT, et al. Older adults with chronic disease: benefits of group-mediated counseling in the promotion of physically active lifestyles. *Health Psychology.* 2003;22(4):414-423.

**201.** Rejeski WJ, Foley KO, Woodard CM, Zaccaro DJ, Berry MJ. Evaluating and understanding performance testing in COPD patients. *Journal of cardiopulmonary rehabilitation.* 2000;20(2):79-88.

**202.** Rejeski WJ, Ip EH, Marsh AP, Miller ME, Farmer DF. Measuring disability in older adults: the International Classification System of Functioning, Disability and Health (ICF) framework. *Geriatrics & gerontology international.* 2008;8(1):48-54.

**203.** Resnick B, Galik E. The reliability and validity of the physical activity survey in long-term care. *Journal of Aging & Physical Activity.* 2007;15(4):439-458.

**204.** Reuben DB, Siu AL. An objective measure of physical function of elderly outpatients. The Physical Performance Test. *Journal of the American Geriatrics Society.* 1990;38(10):1105-1112.

**205.** Ricci NA, Kubota MT, Cordeiro RC. [Agreement between observations on the functional capacity of home care elderly patients]. *Revista de saude publica.* 2005;39(4):655-662.

**206.** Rockwood K, Song X, MacKnight C, et al. A global clinical measure of fitness and frailty in elderly people. *CMAJ Canadian Medical Association Journal.* 2005;173(5):489-495.

**207.** Romano JM, Turner JA, Jensen MP. The Chronic Illness Problem Inventory as a measure of dysfunction in chronic pain patients. *Pain.* 1992;49(1):71-75.

**208.** Rozzini R, Frisoni GB, Ferrucci L, Barbisoni P, Bertozzi B, Trabucchi M. The effect of chronic diseases on physical function. Comparison between activities of daily living scales and the Physical Performance Test. *Age & Ageing.* 1997;26(4):281-287.

**209.** Ruan CM, Haig AJ, Geisser ME, Yamakawa K, Buchholz RL. Functional capacity evaluations in persons with spinal disorders: predicting poor outcomes on the Functional Assessment Screening Test (FAST). *Journal of Occupational Rehabilitation.* 2001;11(2):119-132.

**210.** Ryan CG, Grant PM, Gray H, Newton M, Granat MH. Measuring postural physical activity in people with chronic low back pain. *Journal of Back & Musculoskeletal Rehabilitation.* 2008;21(1):43-50.

**211.** Sallis JF, Haskell WL, Wood PD, et al. Physical activity assessment methodology in the Five-City Project. *American Journal of Epidemiology.* 1985;121(1):91-106.

**212.** Sayers SP, Guralnik JM, Newman AB, Brach JS, Fielding RA. Concordance and discordance between two measures of lower extremity function: 400 meter self-paced walk and SPPB. *Aging-Clinical & Experimental Research.* 2006;18(2):100-106.

**213.** Schag CC, Heinrich RL, Ganz PA. Karnofsky performance status revisited: reliability, validity, and guidelines. *Journal of clinical oncology : official journal of the American Society of Clinical Oncology.* 1984;2(3):187-193.

**214.** Schandry R. Die Entwicklung des Fragebogens für Asthmapatienten (FAP). *Quintessenz.* 1994(Journal Article):55-60.

**215.** Schenkman M, Cutson TM, Kuchibhatla M, Scott BI, Cress ME. Application of the Continuous Scale Physical Functional Performance Test to people with Parkinson disease. *Neurology Report.* 2002;26(3):130-138.

**216.** Schuit AJ, Feskens EJM, Launer LJ, Kromhout D. Physical activity and cognitive decline, the role of the apolipoprotein e4 allele. *Medicine & Science in Sports & Exercise.* 2001;33(5):772-777.

**217.** Schuit AJ, Schouten EG, Westerterp KR, Saris WH. Validity of the Physical Activity Scale for the Elderly (PASE): according to energy expenditure assessed by the doubly labeled water method. *Journal of clinical epidemiology.* 1997;50(5):541-546.

**218.** Schuntermann MF. [The Duke Health Profile (DUKE)]. *Rehabilitation.* 1997;36(1):I-XIV.

**219.** Schwab Rs EAC. Projection Technique for Evaluating Surgery in Parkinson's Disease.

**220.** Schwartz CE, Vollmer T, Lee H. Reliability and validity of two self-report measures of impairment and disability for MS. North American Research Consortium on Multiple Sclerosis Outcomes Study Group. *Neurology.* 1999;52(1):63-70.

**221.** Sclan SG, Reisberg B. Functional assessment staging (FAST) in Alzheimer's disease: reliability, validity, and ordinality. *International Psychogeriatrics.* 1992;4(Suppl 1):55-69.

**222.** Sheikh K, Smith DS, Meade TW, Goldenberg E, Brennan PJ, Kinsella G. Repeatability and validity of a modified activities of daily living (ADL) index in studies of chronic disability. *International rehabilitation medicine.* 1979;1(2):51-58.

**223.** Shin Y, Jang H, Pender NJ. Psychometric evaluation of the Exercise Self-Efficacy Scale among Korean adults with chronic diseases. *Research in nursing & health.* 2001;24(1):68-76.

**224.** Shin Y, Pender NJ, Yun S. Using methodological triangulation for cultural verification of commitment to a plan for exercise scale among Korean adults with chronic diseases. *Research in nursing & health.* 2003;26(4):312-321.

**225.** Sinderby C, Spahija J, Beck J, et al. Diaphragm activation during exercise in chronic obstructive pulmonary disease. *American Journal of Respiratory & Critical Care Medicine.* 2001;163(7):1637-1641.

**226.** Singh PN, Fraser GE, Knutsen SF, Lindsted KD, Bennett HW. Validity of a physical activity questionnaire among African-American Seventh-day Adventists. *Medicine & Science in Sports & Exercise.* 2001;33(3):468-475.

**227.** Skumlien S, Hagelund T, Bjortuft O, Ryg MS. A field test of functional status as performance of activities of daily living in COPD patients. *Respiratory medicine.* 2006;100(2):316-323.

**228.** Stallberg B, Nokela M, Ehrs PO, Hjemdal P, Jonsson EW. Validation of the clinical COPD Questionnaire (CCQ) in primary care. *Health & Quality of Life Outcomes.* 2009;7(Journal Article):26.

**229.** Stavem K, Jodalen H. Reliability and validity of the COOP/WONCA health status measure in patients with chronic obstructive pulmonary disease. *Quality of Life Research.* 2002;11(6):527-533.

**230.** Steele BG, Holt L, Belza B, Ferris S, Lakshminaryan S, Buchner DM. Quantitating physical activity in COPD using a triaxial accelerometer. *Chest.* 2000;117(5):1359-1367.

**231.** Suurmeijer TP, Doeglas DM, Moum T, et al. The Groningen Activity Restriction Scale for measuring disability: its utility in international comparisons. *American Journal of Public Health.* 1994;84(8):1270-1273.

**232.** Syddall HE, Martin HJ, Harwood RH, Cooper C, Aihie Sayer A. The SF-36: a simple, effective measure of mobility-disability for epidemiological studies. *Journal of Nutrition, Health & Aging.* 2009;13(1):57-62.

**233.** Tager IB, Hollenberg M, Satariano WA. Association between self-reported leisure-time physical activity and measures of cardiorespiratory fitness in an elderly population. *American Journal of Epidemiology.* 1998;147(10):921-931.

**234.** Taylor HL, Jacobs DR, Jr., Schucker B, Knudsen J, Leon AS, Debacker G. A questionnaire for the assessment of leisure time physical activities. *Journal of chronic diseases.* 1978;31(12):741-755.

**235.** Tsang AH. *Effectiveness of three strengths of education and exercise on self-efficacy for walking and self-efficacy for managing dyspnea in patients with chronic obstructive pulmonary disease*, University of California, San Francisco; 2000.

**236.** Vallerand AH. Development and testing of the inventory of functional status--chronic pain. *Journal of Pain & Symptom Management.* 1998;15(2):125-133.

**237.** van Helvoort HA, Heijdra YF, de Boer RC, Swinkels A, Thijs HM, Dekhuijzen PN. Six-minute walking-induced systemic inflammation and oxidative stress in muscle-wasted COPD patients. *Chest.* 2007;131(2):439-445.

**238.** van 't Hul A, Gosselink R, Kwakkel G. Constant-load cycle endurance performance: test-retest reliability and validity in patients with COPD. *Journal of cardiopulmonary rehabilitation.* 2003;23(2):143-150.

**239.** Velozo CA, Peterson EW. Developing meaningful Fear of Falling Measures for community dwelling elderly. *American Journal of Physical Medicine & Rehabilitation.* 2001;80(9):662-673.

**240.** Verbunt JA, Westerterp KR, van der Heijden GJ, Seelen HA, Vlaeyen JW, Knottnerus JA. Physical activity in daily life in patients with chronic low back pain. *Archives of Physical Medicine & Rehabilitation.* 2001;82(6):726-730.

**241.** Volpato S, Cavalieri M, Guerra G, et al. Performance-based functional assessment in older hospitalized patients: feasibility and clinical correlates. *Journals of Gerontology Series A-Biological Sciences & Medical Sciences.* 2008;63(12):1393-1398.

**242.** Voorrips LE, Lemmink KA, van Heuvelen MJ, Bult P, van Staveren WA. The physical condition of elderly women differing in habitual physical activity. *Medicine & Science in Sports & Exercise.* 1993;25(10):1152-1157.

**243.** Ware JE, Jr., Sherbourne CD. The MOS 36-item short-form health survey (SF-36). I. Conceptual framework and item selection. *Medical care.* 1992;30(6):473-483.

**244.** Washburn RA, Ficker JL. Physical Activity Scale for the Elderly (PASE): the relationship with activity measured by a portable accelerometer. *Journal of Sports Medicine & Physical Fitness.* 1999;39(4):336-340.

**245.** Webb SM, Prieto L, Badia X, et al. Acromegaly Quality of Life Questionnaire (ACROQOL) a new health-related quality of life questionnaire for patients with acromegaly: development and psychometric properties. *Clinical endocrinology.* 2002;57(2):251-258.

**246.** Webster K, Cella D, Yost K. The Functional Assessment of Chronic Illness Therapy (FACIT) Measurement System: properties, applications, and interpretation. *Health & Quality of Life Outcomes.* 2003;1(Journal Article):79.

**247.** Wellard S. Validation of physical activity measurement for people on dialysis treatment. *EDTNA/ERCA Journal of Renal Care.* 2003;29(3):140-142.

**248.** Whitehurst M, Brown LE, Eidelson SG, D'Angelo A. Functional mobility performance in an elderly population with lumbar spinal stenosis. *Archives of Physical Medicine & Rehabilitation.* 2001;82(4):464-467.

**249.** Winograd CH, Lemsky CM, Nevitt MC, et al. Development of a physical performance and mobility examination. *Journal of the American Geriatrics Society.* 1994;42(7):743-749.

**250.** Yang P, Chen C. Exercise stage and processes of change in patients with chronic obstructive pulmonary disease. *Journal of Nursing Research.* 2005;13(2):97-105.

**251.** Yasuko F. Development, reliability and validity of an ADL gap self-efficacy scale for elderly persons requiring home based nursing care [Japanese]. *Journal of Japan Academy of Nursing Science.* 2002;22(1):23-32.

**252.** Yates JW, Chalmer B, McKegney FP. Evaluation of patients with advanced cancer using the Karnofsky performance status. *Cancer.* 1980;45(8):2220-2224.

**253.** Zanocchi M, Ponzetto M, Francisetti F, et al. [The dependence medical index (DMI): validation and comparison with the activity daily living and the instrumental activity daily living]. *Minerva medica.* 2004;95(2):143-151.

**254.** Zhan S, Cerny FJ, Gibbons WJ, Mador MJ, Wu YW. Development of an unsupported arm exercise test in patients with chronic obstructive pulmonary disease. *Journal of cardiopulmonary rehabilitation.* 2006;26(3):180-187.

**255.** Zimnavoda T, Weinblatt N, Katz N. Validity of the Kohlman Evaluation of Living Skills (KELS) with Israeli elderly individuals living in the community. *Occupational Therapy International.* 2002;9(4):312-325.
